# Accelerated ageing and coronary microvascular dysfunction in chronic heart failure in Tgαq*44 mice

**DOI:** 10.1007/s11357-022-00716-y

**Published:** 2023-01-24

**Authors:** Piotr Berkowicz, Justyna Totoń-Żurańska, Grzegorz Kwiatkowski, Agnieszka Jasztal, Tamás Csípő, Kamil Kus, Urszula Tyrankiewicz, Anna Orzyłowska, Paweł Wołkow, Attila Tóth, Stefan Chlopicki

**Affiliations:** 1 Jagiellonian Centre for Experimental Therapeutics (JCET), Jagiellonian University, Krakow, Poland; 2 Centre for Medical Genomics OMICRON, Jagiellonian University Medical College, Krakow, Poland; 3 Division of Clinical Physiology, Department of Cardiology, Faculty of Medicine, University of Debrecen, Debrecen, Hungary; 4 Department of Public Health, Faculty of Medicine, Semmelweis University, Budapest, Hungary; 5 Department of Neurosurgery and Paediatric Neurosurgery, Medical University of Lublin, Lublin, Poland; 6 Faculty of Medicine, Chair of Pharmacology, Jagiellonian University Medical College, Krakow, Poland

**Keywords:** Ageing, Coronary microvascular dysfunction, Heart failure, Tgαq*44 mice

## Abstract

Age represents a major risk factor in heart failure (HF). However, the mechanisms linking ageing and HF are not clear. We aimed to identify the functional, morphological and transcriptomic changes that could be attributed to cardiac ageing in a model of slowly progressing HF in Tgαq*44 mice in reference to the cardiac ageing process in FVB mice. In FVB mice, ageing resulted in the impairment of diastolic cardiac function and in basal coronary flow (CF), perivascular and interstitial fibrosis without changes in the cardiac activity of angiotensin-converting enzyme (ACE) or aldosterone plasma concentration. In Tgαq*44 mice, HF progression was featured by the impairment of systolic and diastolic cardiac function and in basal CF that was associated with a distinct rearrangement of the capillary architecture, pronounced perivascular and interstitial fibrosis, progressive activation of cardiac ACE and systemic angiotensin-aldosterone-dependent pathways. Interestingly, cardiac ageing genes and processes were represented in Tgαq*44 mice not only in late but also in early phases of HF, as evidenced by cardiac transcriptome analysis. Thirty-four genes and 8 biological processes, identified as being ageing related, occurred early and persisted along HF progression in Tgαq*44 mice and were mostly associated with extracellular matrix remodelling and fibrosis compatible with perivascular fibrosis resulting in coronary microvascular dysfunction (CMD) in Tgαq*44 mice. In conclusion, accelerated and persistent cardiac ageing contributes to the pathophysiology of chronic HF in Tgαq*44 mice. In particular, prominent perivascular fibrosis of microcirculation resulting in CMD represents an accelerated cardiac ageing phenotype that requires targeted treatment in chronic HF.

**Supplementary Information:**

The online version contains supplementary material available at 10.1007/s11357-022-00716-y.

## Introduction

The global population is rapidly ageing, which leads to novel social and economic challenges worldwide [1]. Age is considered a major risk factor for the development of cardiovascular diseases [2], such as heart failure (HF), which is a leading cause of mortality globally [3]. In fact, HF is a disease characterised by increasing morbidity with ageing, with a prevalence of ~ 1% in the 40- to 59-year age group and increasing up to ~ 12% in those aged over 80 years [4].

Multiple risk factors and pathomechanisms are involved in chronic HF. Because the progression of HF is linked to excessive activation of the adrenergic nervous system and the renin-angiotensin aldosterone system (RAAS), the neurohormonal blockade represents a major therapeutic strategy in HF [5]. Interestingly, there is recent evidence pointing out that coronary microvascular dysfunction (CMD) contributes to the pathophysiology of HF [6]. In particular, previous studies highlighted an increased prevalence and importance of CMD in the pathophysiology of HF with preserved ejection fraction (HFpEF) [7–11]—the variant of HF commonly observed in the elderly that is not particularly responsive to the treatment based on the neurohormonal blockade compared with HF with reduced ejection fraction (HFrEF) [12, 13].

CMD refers to structural and functional alterations in coronary microcirculation [14, 15]. Structurally, CMD involves luminal narrowing of arterioles and capillaries, perivascular fibrosis and capillary rarefaction [16]. Functionally, CMD is linked to impaired vasodilation of coronary microvessels [17], involving alterations in the endothelium-dependent or endothelial-independent function of coronary microcirculation [18]. Functional impairment of coronary microvascular function may be triggered by low-grade systemic inflammation [19, 20], reduction of nitric oxide (NO) bioavailability, increased levels of vasoconstriction mediators [21], increased oxidative stress, increased leukocyte infiltration, neurohormonal activation, microvascular wall barrier dysfunction and altered vascular endothelial permeability [22].

It is well accepted that CMD plays an important role in the pathophysiology of myocardial ischemia [23]. Indeed, abnormalities in coronary microcirculation can cause myocardial ischemia and angina [24] even without the stenosis of larger arteries. It was reported that as much as 65% of women and 32% of men [25] display stable angina pectoris without obstructive coronary artery disease (CAD) [23]. Accordingly, CMD has an important prognostic value in angina with and without obstructive CAD [26], as well as in myocardial infarction [27]. Interestingly, CMD also has prognostic value in non-ischemic cardiomyopathy [28], suggesting a more important role of CMD in HF that is not limited to ischaemic heart disease [29].

Given the increasing importance of CMD in clinical cardiology across the spectrum of cardiovascular diseases [17], CMD diagnostics have recently advanced and are increasingly performed based on invasive (coronary angiography, intracoronary Doppler flow wire, intracoronary thermodilution) or non-invasive (transthoracic Doppler echocardiography, positron emission tomography, cardiovascular magnetic resonance imaging (MRI)) methods [30, 31]. However, the pathomechanisms of CMD in chronic HF still remain elusive.

Importantly, many of CMD’s pathomechanisms may be associated with vascular ageing [32, 33]. Indeed, age-related microvascular alteration in the heart was linked to increased oxidative stress, decreased NO bioavailability, inflammation and perivascular fibrosis [19, 34], causing impaired coronary microvascular function manifested by reduction of coronary flow (CF) reserve [35]. Of note, many other pathophysiological features of HF beyond CMD are shared with cardiac ageing, including left ventricular diastolic dysfunction, left ventricular hypertrophy, loss of cardiomyocytes, fibrosis, extracellular matrix remodelling, mitochondrial dysfunction and changes in calcium signalling [35–39]. Accordingly, multiple facets of cardiac ageing may play an important role in HF progression, but the contribution of these ageing-related mechanisms to the pathophysiology of HF is not well defined.

In the present work, in an effort to better understand the role of cardiac ageing in the pathophysiology of chronic HF, we took advantage of a unique murine model of slowly developing HF, initially developed by Mende et al. [40]. In Tgαq*44 mice, the progression of HF to the end-stage phenotype is prolonged and occurs at the age of 12–14 months [41, 42]. At this age, the features of cardiac ageing are present in mice [43] and, thus, could well contribute to HF pathophysiology, validating the choice of this unique model of chronic HF for our study. Our approach was to characterise the age-related cardiac phenotype on functional, morphological and transcriptomic levels in wild-type FVB mice along ageing and then to identify possible age-related changes in the heart in Tgαq*44 mice along the progression of HF. Our results suggest that in Tgαq*44 mice, there is accelerated cardiac ageing, and it is exemplified by robust perivascular fibrosis and extracellular matrix remodelling in coronary microvasculature resulting in CMD. We claim that these changes contribute to the pathophysiology of chronic HF in Tgαq*44 mice and, thus, represent an intersection of cardiac ageing and HF pathophysiology.

## Materials and methods

### Animals

Tgαq*44 and FVB (wild-type) mice were bred in the Animal Laboratory of the Medical Research Centre of the Polish Academy of Sciences (Warsaw, Poland). All animal procedures conformed to the Guide for the Care and Use of Laboratory Animals published by the National Institutes of Health (NIH Publication No. 85–23, revised 1996) as well as to the local Ethical Committee on Animal Experiments in Krakow. Mice were fed a standard chow diet, given water ad libitum and kept in pathogen-free conditions (22–25 °C, 45–65% humidity, 12 h light/12 h dark cycle). Female mice showing early (4–6 months), transition (8–10 months) and end-stage (12–14 months) HF development in Tgαq*44 mice [42] and age-matched control FVB mice were used.

## MRI measurements

### MRI data acquisition

All experiments were performed using a dedicated, small-animal 9.4 T MRI scanner (Bruker BioSpin, Ettlingen, Germany) equipped with a 36 mm quadrature coil. Throughout the measurements, the animals were placed in a supine position in a dedicated cradle and anaesthetised with 1.7% isoflurane in a 1:2 oxygen-air mixture delivered via a nose cone. Electrocardiogram (ECG), respiration and body temperature maintained at 37 °C by water heating were monitored (SA Instruments). To evaluate the haemodynamics of the left ventricle (LV), bright-blood cine images were collated in 6–7 contiguous slices covering the entire ventricle volume using a flow-compensated, prospectively gated gradient-echo FLASH sequence with the following parameters: FOV 30 × 30 mm^2^, acquisition matrix: 192 × 192, TE/TR = 2.3/5 ms, slice thickness = 1 mm, number of averages = 4, flip angle = 11°. Depending on the heart rate (HR), between 22 and 24 cine frames were acquired. The filling and ejection rates of LV were obtained with a high frame rate, retrospectively gated cine FLASH sequence (IgFLASH) in a mid-ventricular, short-axis slice. The following acquisition parameters were used: FOV 30 × 30 mm^2^, acquisition matrix: 128 × 128, TE/TR = 1.3/4.2 ms, slice thickness = 1 mm, number of repetitions = 1200, flip angle = 11°. The data were reconstructed to 60 frames per cardiac cycle using a vendor-provided macro (ParaVision 6.0.1, Bruker BioSpin, Ettlingen, Germany). Tagged cine images were obtained using a double-gated FLASH sequence (FOV 30 × 30 mm^2^, acquisition matrix: 192 × 192, TE/TR = 1.5/4.8 ms, slice thickness = 1 mm, number of repetitions = 16, flip angle = 11°, 20–25 frames) with a Spatial Modulation of Magnetization (SPAMM) module for tag generation (square tags: line thickness 0.2 mm, span 0.6 mm).

### Data analysis

A time-volume curve (TVC) was calculated from LV volumes (including papillary muscles), as assessed using short-axis slice-by-slice semiautomatic segmentation (Segment; Medviso). End-systolic (ESV) and end-diastolic (EDV) volumes, stroke volume (SV), ejection fraction (EF), cardiac output (CO) and cardiac index (CI) were assessed from the TVC. A piecewise linear regression (PLR) implemented in MATLAB (MathWorks), as described previously [44], was used to obtain the ejection rate (ER) and filling rate (FR) with slopes of segments fitted by PLR normalised to the individual SV and R-R intervals. Durations of ejection time (ET), isovolumetric relaxation time (IVRT), filling time (FT) and isovolumetric contraction time (IVCT) were taken from the PLR model and normalised to the R-R interval. For the tagged image analysis, the peak-combination harmonic phase (HARP) algorithm was implemented in MATLAB. The midlevel HARP maps were used to compute radial (Err) and circumferential (Ecc) strains (peak systolic (Es), end-systolic (Ees), maximum postsystolic (Epost)) and strain rates (systolic (SRmax), early diastolic (SRe), atrial diastolic (SRa)), as well as early/atrial diastolic strain ratio (SRe/a), systolic stretch (SS), postsystolic strain index (PSI = (Epost-Ees)/Emax) and time-to-peak strain index (TpeakSI = Tpeak/RR) as described in the study of Tyrankiewicz U. et al. [42]. Strains were assessed in eight consistent segments encircling the myocardial cross-section and then averaged.

## Doppler blood flow velocity mapping

Peak blood flow velocity (PVF) of basal CF was monitored with the usage of a Doppler flow velocity system (Indus Instruments, Scintica Instrumentation). Mice were anaesthetised with isoflurane (Aerrane; Baxter Sp. z o. o., 1.5 vol%) in an oxygen mixture. Mice were placed in a supine position on the temperature‐controlled board and monitored via ECG. The temperature gauge was placed rectally to control the body temperature maintained at 37 °C. A 20-MHz probe oriented almost parallel to the board surface and aimed towards the upper mid‐chest in the area of the heart was used to find the characteristic blood flow velocity pattern of CF in the left anterior descending artery. PVF of basal CF was collected during anaesthesia set by 1.5% isoflurane. Speckles for basal CF velocity were analysed by Doppler signal processing workstation (version 1.625, Indus Instruments).

## RNA isolation and RNA-Seq analysis

Total RNA was extracted from cardiac tissues (*i.e.,* apex of the heart) using an RNeasy Mini Kit (Qiagen) with DNAse treatment. RNA quality was controlled on TapeStation (Agilent). mRNA libraries were prepared with a Sense mRNA-Seq Library prep Kit v2 (Lexogen) according to the manufacturer’s protocol. Briefly, after normalisation, 1000 ng of RNA was denatured, polyA-selected on magnetic beads and purified. After reverse transcription, second strand synthesis was performed. cDNA was amplified and indexed in 13 PCR cycles. Pooled libraries were sequenced on NextSeq (Illumina) using a NextSeq 500/550 High Output Kit v2.0 at 1.8 pM final concentration. The 50 cardiac RNA samples were sequenced in three runs.

## Transcriptomic analyses

Adaptor sequences, primers and poly-A tails were trimmed from sequencing raw reads by Cutadapt [45], and the quality of the raw reads was checked by FastQC (https://www.bioinformatics.babraham.ac.uk/projects/fastqc/). Further, the raw reads were mapped towards the mouse reference genome using Star (approximately 78% of raw reads were uniquely mapped into the reference genome) [46]. The HTSeq software was used to count reads that mapped to the exons of the coding genes [47]. Normalisation of the count data was performed using the TMM method [48]. 30.01% of 54 532 genes in the reference mouse genome (i.e., 16 367 genes) met the applying coverage-based filtering criteria of cpm > 0.5 in more than 3 samples in a group. Analysis of differentially expressed genes (DEGs) was performed using the Limma-Voom software [49]. Analyses and plots were generated in R environment (https://www.r-project.org/) or GraphPad Prism software (version 8.3.1). The following filtering criteria for DEGs were used: |log fold change|> 1, adjusted P-value < 0.05 for Benjamini and Hochberg adjusting method. 84% of genes (*i.e.,* 13 778 genes) out of 16 367 genes that met the applying coverage-based filtering criteria were annotated to biological processes from the Gene Ontology database. For testing over-representations of the biological processes, Fisher’s exact test for count data was used. To improve the reliability of the Fisher’s test results, the Benjamini and Hochberg method was applied for *P*-value correction. Biological processes were considered over-represented when the *P*-value was < 0.05. Principal component analysis (PCA) was applied to the standardised dataset with prior rank normalisation and used to characterise dominant directions of maximal sample variation of cardiac ageing genes in the ageing heart and developing HF transcriptome.

### Methodological approach

Two separate differential gene expression analyses were performed to assess changes related to (1) the cardiac ageing process (4-month-old FVB mice compared to older ones—FVB vs. FVB analysis) and (2) HF development along the cardiac ageing process (Tgαq*44 mice compared to age-matched FVB mice—Tgαq*44 vs. FVB analysis). Within the first analysis, the DEGs and over-represented processes were classified into two different categories according to the propagation of ageing: ‘*genes* and *processes of ageing heart*’ (DEGs and over-represented biological processes found in 14-month-old FVB mice as well as at least in one of the younger group) and ‘*genes* and *processes of aged heart*’ (found only in 14-month-old FVB mice but not in younger ones).

## Transmission electron microscopy (TEM)

Immediately after sacrificing the mice, cardiac tissue samples from the region of the papillary muscle and free wall of the LV were inserted in a solution of 2% paraformaldehyde and 2.5% glutaraldehyde in 0.1 M cacodylate buffer of pH 7.4 for 24 h at 4 °C. Further, cardiac tissue was postfixed in 1% osmium tetroxide for 1 h at room temperature. Dehydration was performed by incubating the sample in increasing ethanol concentrations: 50% (10 min), 70% (40 min), 90% (10 min), 96% (10 min) and 2 × 100% (each 10 min), then in a mixture of 100% ethanol:100% propylene oxide in a 1:1 ratio (10 min) and in 2 × 100% propylene oxide (each 15 min). During dehydration, the tissue was stained with 1% uranyl acetate in 70% ethanol (40 min). Finally, cardiac tissue was embedded in the Epon resin. Ultrathin Sects. (60 nm) were collected on grids and poststained with uranyl acetate and Reynold’s lead citrate. Electron micrographs were obtained with a Morada G2 camera on a JEM 1400 transmission electron microscope at 80 kV (JEOL Co., Japan) in the Laboratory of Electron Microscopy, Nencki Institute of Experimental Biology of Polish Academy of Sciences, Warsaw, Poland.

## Determination of steroid hormones in plasma

The concentrations of 17 steroid hormones in plasma (Fig. S1) were evaluated on the basis of liquid chromatography with tandem mass spectrometry (LC–MS/MS) measurements using a commercially available StereoIDQ® kit (Biocrates Life Sciences AG, Innsbruck, Austria) according to the kit manufacturer’s instructions and a developed LC–MS/MS method. Chromatographic separation was performed using a steroid-specific high-performance liquid chromatography (HPLC) column (delivered with the kit) and precolumn. The HPLC system was a UFLC Nexera (Shimadzu, Kyoto, Japan). A QTrap 5500® triple quadrupole mass spectrometer (Sciex, Toronto, Canada) equipped with an ESI-Turbo V source operating in positive ionisation mode and controlled by the Analyst 1.6.3 software (Sciex, Toronto, Canada) was used for detection. Multiple reaction monitoring (MRM) was applied for highly selective and sensitive detection of the analytes. The elution of the steroid hormones from the samples was carried out according to a protocol provided with the StereoIDQ® kit in two subsequent SPE-based steps (Supplementary Table S1). Quantification of metabolite concentration and quality assessment were performed with the MetIQ software (Biocrates Life Sciences, Innsbruck, Austria).

## Isolated murine heart preparation

Mice were anaesthetised with ketamine (100 mg/kg of body weight) and xylazine (10 mg/kg of body weight). The hearts were excised from Tgαq*44 and age-matched FVB mice (4- to 6-, 8-, 12-, 14- to 15-month-old), immersed in ice-cold Krebs–Henseleit buffer, mounted by the ascending aorta within 2 min to the heart apparatus (IH-SR, Type 844, Hugo Sachs Electronics (HSE), Germany) and perfused retrogradely according to the Langendorff method at a perfusion pressure of 100 mmHg. The Krebs–Henseleit buffer was composed of (mM): NaCl 118.00, KCl 4.70, CaCl_2_ 2.52, MgSO_4_ 1.64, NaHCO_3_ 24.88, KH_2_PO_4_ 1.18, glucose 10.00, sodium pyruvate 2.00 and EDTA 0.50 and was used for heart perfusions. In isolated hearts from 14- to 15-month-old Tgαq*44 and FVB mice (depicted in the figure as 14-month-old mice), the hearts were perfused with modified Krebs buffer composed of (mM): NaCl 118.00, KCl 4.70, CaCl_2_ 2.52, MgSO_4_ 1.64, NaHCO_3_ 24.88, KH_2_PO_4_ 1.18, glucose 5.00, sodium pyruvate 0.50, lactate 1.00, leucine 0.25, isoleucine 0.25, valine 0.30, l-glutamic acid 0.25, octanoic acid 0.50, 3-hydroxybutyric acid 0.40, l-carnitine 0.05 and EDTA 0.50. The pH value of the Krebs–Henseleit buffer was equilibrated to approximately 7.4 by bubbling with a gas mixture of 95% O_2_/5% CO_2_ and perfusion of the buffer through the oxygenator (Fiber Oxygenator Type D150, Harvard Apparatus). The perfusate was maintained at 37 °C. CF changes were monitored using an Ultrasonic flowmeter (Transit Time Flowmeter TTFM Type 700, HSE) and analysed using the ISOHEART software (version: 1.1.1.202 (32)). Vasodilatation was determined by subtracting CF at baseline flow from a peak of CF during reactive hyperaemia or the addition of bradykinin and presented as the increase in CF (ΔCF). ΔCF was normalised to the ventricles mass. CF reserve in the isolated perfused heart was assessed by dividing the maximum CF in response to 30-s occlusion of CF by baseline CF.

### Experimental protocol of ex vivo perfused murine heart

After heart mounting and 15 min of the stabilisation period, hearts were paced with 450 beats per minute. Subsequently, 30-s occlusion of CF, followed by a bolus injection of bradykinin to the perfusion line, was performed. Basal CF was assessed 15 min after the initiation of cardiac perfusion.

## Blood morphology

Blood samples were drawn from the right heart ventricle into a syringe with coagulant (heparin, 1000 I.U. per ml of blood). The blood cell count was assessed using an animal blood counter (ABC Vet, Horiba, Germany).

## ACE activity

ACE activity was measured in plasma and cardiac ventricular tissue by methods previously described [50, 51]. In brief, tissue samples were homogenised in 100.0 mM Tris·HCl buffer via a Praecellys Evolution bead mill homogeniser. Homogenised samples were then diluted to a protein concentration of 1 mg/mL. Plasma samples (6 μL in 200 μL) or cardiac homogenates (final protein concentration: 0.1 mg/mL) were placed into individual wells of a 96 well plate (Type 3651, Corning, Corning, NY, USA). ACE substrate containing (end concentration: 100.0 mM Tris·HCl, 15.0 μM Abz-FRK(Dnp)P, 50.0 mM NaCl and 10.0 μM ZnCl_2_ at pH 7.0), pre-warmed buffer was then added to each well. ACE substrate (Abz-FRK(Dnp)P) was synthetised by Peptide 2.0 (Chantilly, VA, USA), and all other chemicals were obtained from Sigma-Aldrich (St. Louis, MO, USA). Kinetic measurement of ACE activity in plasma and cardiac homogenates was performed using a fluorescence microplate reader (Synergy 4™, Biotek, Santa Clara, CA, USA). Excitation was set to 340 nm, and emissions were read at 420 nm. The measured fluorescence values were then plotted as a function of time and the linear slope was recorded. The slope was then used to calculate ACE activity with the following formula: ACE activity = ([slope]/[fluorescence intensity of 1 µmol converted substrate]) × *Y*, where *Y* is the dilution factor (33.33, in the case of plasma, and 10 in the case of tissue samples). ACE activity in plasma was presented as converted ACE substrate in U/L and in cardiac homogenates as converted ACE substrate in U/g.

## Histological evaluation of cardiac tissue

Hearts were exited from the mice and fixed in 4% buffered formalin. The fixed hearts were cut in a perpendicular plane to their long axis at half of their length. The part of the heart with the apex was further processed according to paraffin method [52]. The tissue was embedded in paraffin blocks in a position, which enabled to get transverse sections through the heart on the level of papillary muscles with still clearly visible lumens of both cardiac chambers. The blocks with cardiac tissue were cut into 5 µm thick sections and placed by three sections per microscope slide on four consecutive microscope slides. Each of this four microscope slides were stained by a different staining technique. All histological stainings were performed accordingly to standardised methods. Each staining method were performed simultaneously in the same process using the same reagent solutions in an automatic stainer. Histological preparations prepared in this manner were considered a basis for obtaining images for quantitative calculations and qualitative analyses.

### Stainings

Sections of cardiac tissue were stained with haematoxylin and eosin (HE) for general histology and with Picro Sirius Red (PSR) for collagen deposition. The microcoronary architecture and capillary density were characterised by immunohistological staining of glycocalyx with lectin and DAB as the chromogen. Extracellular matrix proteins rich of sugar residues and their colocalisation with the network of blood vessels were visualised by staining with lectin, DAB as the chromogen and periodic acid–Schiff (PAS) with picric acid as contra staining.

### Histologic visualisation and analysis

Cardiac tissue sections were visualised using a standard light microscope Olympus BX51 with VS-ASW 2.6 software (Olympus Corporation, Tokyo, Japan). Quantitative and qualitative evaluations of heart samples were performed under 100, 200 and × 400 magnification. The algorithm for evaluating the quantity of stromal cellularity stained by HE in cardiac tissue (under × 100 magnification) was developed by using the ilastik segmentation toolkit and Fiji ImageJ software (NIH, Bethesda, MD, United States). One full section through the heart in the middle of its length stained by PSR and evaluated under a magnification of × 100 was used to assess cardiac fibrosis for each mice. The ilastik and ImageJ software were used to create an algorithm that was used for counting surface area of the yellow-coloured myocardium and contrast-red-coloured collagen. The degree of fibrosis was calculated as % of collagen of the entire cardiac tissue section (collagen + myocardium). 10 random images for each mice of lectin-stained cardiac cross-sections from the area of papillary muscles were used to evaluate capillary density and the smallest averaged distances between oblique and transverse cardiac capillaries (under × 200 magnification) by using Columbus software. The images obtained from these areas of the myocardium represented mainly transverse sections through the muscle fibres and mostly the cross-sections through the capillaries. These cross-sections of myocardium were considered appropriate for the assessment of the spatial arrangement of capillary network and distances between oblique and transverse capillaries. The algorithm for capillary density counted the area of capillary cross sections (in various spatial configurations: longitudinal, transverse and oblique) and normalised these values to the tissue area. The algorithm also identified capillaries with visible lumen and calculates the lumen area of such vessels. Extracellular matrix proteins with neutral sugars (GAGs, glycoproteins) were evaluated using colour inversion imaging (under × 400 magnification) in transverse sections of the heart in the area of papillary muscles and in longitudinal section of the heart muscle (lectin staining with paS and picric acid) obtained from the free wall of the left ventricular chamber.

## Statistics

Statistical analyses were performed using GraphPad Prism software (version 8.3.1). Data are presented as the mean ± SD. The normality of the data distribution was assessed using the Shapiro–Wilk test and the homogeneity of variance with the Fisher test. Statistical significance was evaluated by Student’s *t* test or Mann–Whitney test between Tgαq*44 and age-matched FVB mice, between 4- and 12-month-old Tgαq*44 mice for evaluating cardiac haemodynamic parameters. One-way ANOVA with a post hoc Tukey’s test or Kruskal–Wallis test with a post hoc Dunn’s test was used to compare older animals (6-, 8-, 10-, 12-, 14-month-old) with 4-month-old animals. Differences were considered to be significant at *P* < 0.05.

## Results

### Alterations in CF in HF in Tgαq*44 mice compared to age-matched FVB mice

**Fig. 1:**
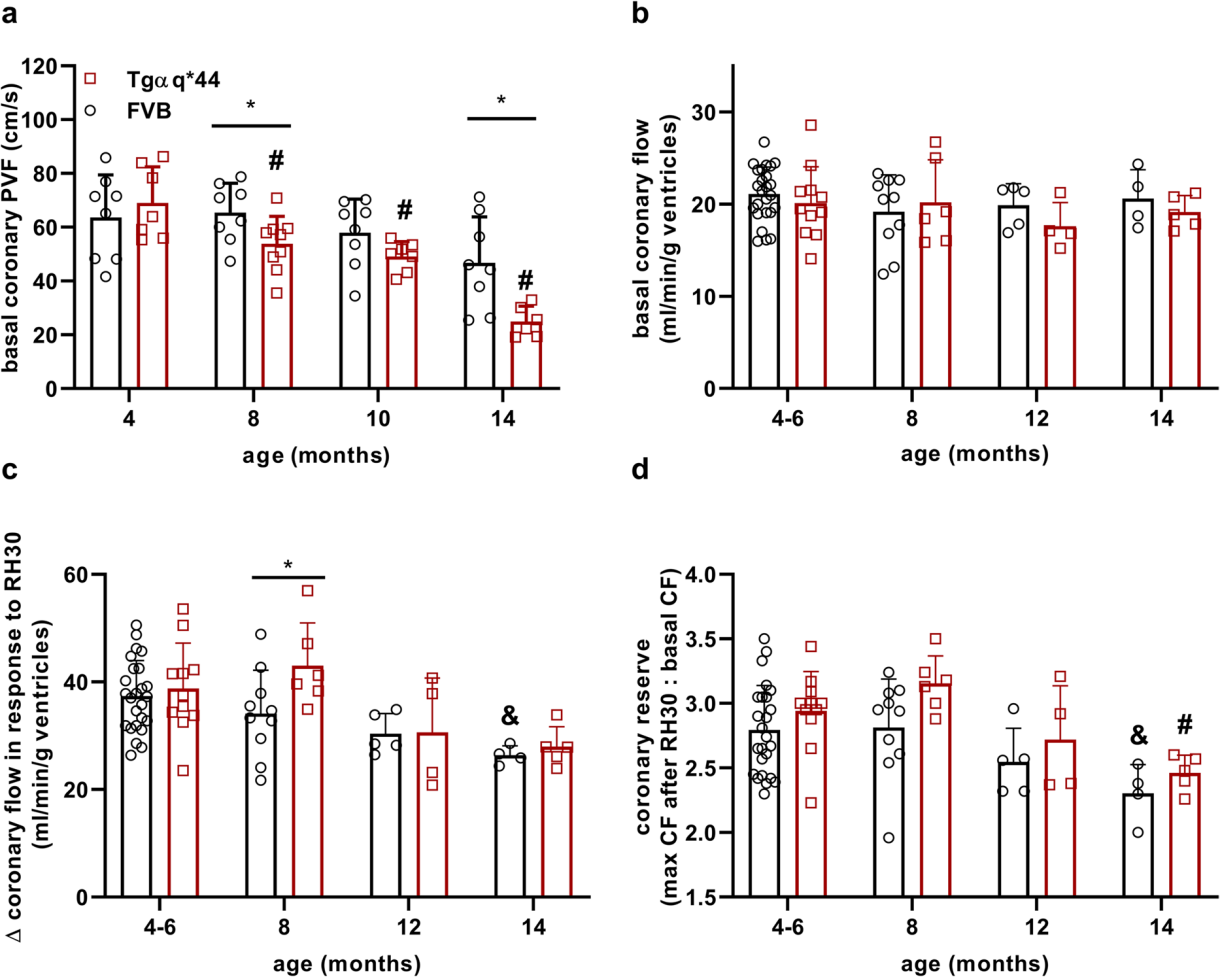
Progressive deterioration of basal CF in the course of HF development in Tgαq*44 mice compared with age-related changes in FVB mice. Basal CF velocity measured in vivo by Doppler ultrasound (**a**), basal CF, increase in CF in response to 30-s occlusion of CF and coronary reserve measured ex vivo in isolated perfused hearts (**b**–**d**). Coronary reserve was assessed either as the relative increase in CF (**c**) or as a ratio of the maximum CF in response to 30-s occlusion of CF to the baseline CF value (**d**). The data are presented as the mean ± SD; *n* = 4–25, *P < 0.05 for Tgαq*44 mice vs. age-matched FVB mice (Student’s *t* test or Mann–Whitney); ^#^*P* < 0.05 for older Tgαq*44 mice vs. 4-month-old Tgαq*44 mice; ^&^*P* < 0.05 for older FVB mice vs. 4-month-old FVB mice (one-way ANOVA with post hoc Tukey’s test). Legend: PVF—peak blood velocity flow, CF – coronary flow, RH30 – reactive hyperaemia induced by 30-s occlusion of CF

In 14-month-old FVB mice, basal blood flow velocity in the coronary circulation measured in vivo by Doppler ultrasound based on PVF measurements tended to be lower compared to 4-month-old FVB mice (Fig. 1a). In 16-month-old FVB mice the difference reached statistical significance (63.64 ± 15.84 cm/s vs. 41.00 ± 7.79 cm/s in 4- and 16-month-old FVB mice, *n* = 7–8, respectively). In contrast, in Tgαq*44 mice, basal CF velocity progressively decreased in the course of HF development, reaching a significantly lower value of basal coronary PVF in 8- and 14-month-old Tgαq*44 mice compared to age-matched FVB mice and to 4-month-old Tgαq*44 mice (Fig. 1a). In contrast to in vivo measurements, in the isolated retrogradely perfused heart according to Langendorff, basal CF was similar in Tgαq*44 and FVB hearts (Fig. 1b). Notably, the magnitude of reactive hyperaemia (RH) induced by 30-s of coronary occlusion was higher in isolated hearts from 8-month-old Tgαq*44 mice compared to age-matched FVB mice (Fig. 1c). There also was a progressive age-dependent decrease in the magnitude of RH in Tgαq*44 and FVB mice (Fig. 1c) resulting in diminished coronary reserve in 14-month-old Tgαq*44 and 14-month-old FVB mice compared to 4-month-old respective counterparts (Fig. 1d). Of note, despite higher RH in isolated hearts in 8-month-old Tgαq*44 mice, the reactivity of bradykinin was comparable to that in FVB hearts (Fig. S2).

### Alterations in systolic and diastolic cardiac function in HF in Tgαq*44 mice compared to age-matched FVB mice

MRI-based measurements demonstrated that 16-month-old FVB mice displayed diastolic cardiac function impairment compared to 4-month-old FVB mice, as evidenced by a decrease in E/A ratio and FR (Table 1) with no significant changes in strain values (Table 2). In turn, even in 4-month-old Tgαq*44 mice, global heart function was impaired in both systolic and diastolic phases (Table 1). SV, EF, CO and FT decreased, whereas ESV, FR and IVCT increased in 4-month-old Tgαq*44 mice compared to age-matched FVB mice (Table 1). Furthermore, the radial and circumferential strains analysis showed that in 4-month-old Tgαq*44 mice Es, Ees, Epost and SRmax values decreased compared to 4-month-old FVB mice (Table 2). Eventually, in 12-month-old Tgαq*44 mice, global heart function was severely impaired, as evidenced by decreased SV, EF, CO, CI and ET compared to 4-month-old Tgαq*44 mice and age-matched FVB mice (Table 1). MRI-based measurements confirmed the previous results describing cardiac performance of FVB and Tgαq*44 mice [41, 42] but extended them by showing altered early to atrial mitral inflow ratio (E/A ratio) and decreased FR, suggestive of deterioration in diastolic cardiac function in 16-month-old FVB mice. However, systolic cardiac function in 16-month-old FVB mice was fully preserved in contrast to the impairment of both diastolic and systolic cardiac function in young 4-month-old Tgαq*44 mice that further deteriorated in older Tgαq*44 mice.

**Table 1:** Haemodynamic parameters of cardiac function in 4-, 12- and 16-month-old FVB mice and 4- and 12-month-old Tgαq*44 mice

	FVB 4 months	FVB 12 months	FVB 16 months	Tgαq*44 4 months	Tgαq*44 12 months
Body mass [g]	26.1 ± 1.8	**30.5 ± 0.9**^&^	**32.1 ± 6.3**^&^	26.1 ± 2.8	26.1 ± 1.4^*^
HR [bpm]	487 ± 53	471 ± 20	440 ± 46	524 ± 15.0	**377 ± 33**^#*^
LV mass [mg]	71.0 ± 5.9	**82.2 ± 3.7**^&^	**86.0 ± 10.1**^&^	79.4 ± 4.7^*^	**71.6 ± 7.1**^#*^
ESV [μl]	15.1 ± 5.6	16.7 ± 4.1	14.3 ± 4.6	27.1 ± 4.9^*^	23.4 ± 8.0
EDV [μl]	53.0 ± 5.1	57.0 ± 6.9	53.0 ± 7.8	57.2 ± 7.2	**41.7 ± 10.6**^#*^
SV [μl]	37.9 ± 4.9	40.3 ± 3.3	38.7 ± 4.4	30.1 ± 3.0^*^	**18.4 ± 3.5**^#*^
EF [%]	71.9 ± 9.1	71.0 ± 4.2	73.4 ± 5.1	54.3 ± 2.4^*^	**44.8 ± 6.0**^#*^
CO [ml/min]	18.3 ± 1.7	19.0 ± 1.8	17.1 ± 2.6	15.8 ± 1.7^*^	**6.9 ± 1.5**^#*^
CI [μl/min/cm^2^]	212.3 ± 19.0	197.9 ± 21.7	**174.1 ± 30.8**^**&**^	182.6 ± 12.8^*^	**80.4 ± 17.3**^#*^
ER [LV/R-R]	317.2 ± 38.6	307.2 ± 22.0	313.8 ± 24.8	290.0 ± 18.6	376.3 ± 81.2
FR [LV/R-R]	472.9 ± 73.0	367.4 ± 86.2	**352.9 ± 95.1**^&^	573.8 ± 70.1^*^	668.1 ± 138.0^*^
ET [% R-R]	28.8 ± 5.2	29.4 ± 4.2	27.9 ± 3.1	34.0 ± 2.9	**19.4 ± 4.2**^#*^
FT [% R-R]	33.2 ± 11.7	38.1 ± 4.2	38.8 ± 6.0	18.9 ± 6.1^*^	20.0 ± 3.3^*^
IVRT [% R-R]	24.0 ± 4.8	24.5 ± 1.9	23.4 ± 3.6	21.9 ± 2.1	19.7 ± 4.4
IVCT [% R-R]	9.9 ± 3.2	9.7 ± 1.7	10.0 ± 3.1	25.7 ± 5.4^*^	33.9 ± 7.9^*^
E/A ratio	2.2 ± 0.3	2.0 ± 0.4	**1.7 ± 0.4**^&^	/	/

The data are presented as the mean ± SD; *n* = 5–9, ^&^*P* < 0.05 for 12- or 16- vs. 4-month-old FVB mice (one-way ANOVA with post hoc Tukey’s test or Kruskal–Wallis test with post hoc Dunn’s test); *P < 0.05 for Tgαq*44 mice vs. age-matched FVB mice; ^#^*P* < 0.05 for 12- vs. 4-month-old Tgαq*44 mice (Student’s *t* test or Mann–Whitney test). Legend: *HR*, heart rate; *LV*, left ventricle; *ESV*, end systolic volume; *EDV*, end diastolic volume; *SV*, stroke volume; *EF*, ejection fraction; *CO*, cardiac output; *CI*, cardiac index; *ER*, ejection rate; *FR*, filling rate; *ET*, ejection time; *FT*, filling time; *IVRT*, isovolumetric relaxation time; *IVCT*, isovolumetric contraction time; *E/A ratio*, early (E) to late (atrial—A) ventricular filling velocity; *R-R*, the time elapsed between two successive R-waves of the QRS signal on the electrocardiogram

**Table 2:** Cardiac strain values in 4-, 12- and 16-month-old FVB mice and 4-month-old Tgαq*44 mice

	Radial strain (Err)				Circumferential strain (Ecc)			
	FVB 4 m	FVB 12 m	FVB 16 m	Tgαq*44 4 m	FVB 4 m	FVB 12 m	FVB 16 m	Tgαq*44 4 m
Es [%]	20.1 ± 2.9	20.6 ± 1.9	20.9 ± 1.5	14.4 ± 1.3^*^	− 16.8 ± 1.7	− 17.8 ± 0.3	− 17.1 ± 1.6	− 12.7 ± 1.4^*^
Ees [%]	20.1 ± 2.9	21.0 ± 2.0	21.6 ± 1.7	16.0 ± 1.4^*^	− 17.7 ± 2.0	− 18.9 ± 0.7	− 17.6 ± 1.6	− 13.4 ± 1.2^*^
Epost [%]	19.4 ± 2.5	19.7 ± 1.6	19.1 ± 4.6	15.0 ± 0.9^*^	− 16.8 ± 2.0	− 17.2 ± 0.5	− 17.2 ± 1.7	− 12.1 ± 1.3^*^
Tpeak	0.41 ± 0.04	0.42 ± 0.05	0.40 ± 0.05	0.51 ± 0.04^*^	0.43 ± 0.06	0.41 ± 0.02	0.41 ± 0.05	0.51 ± 0.03^*^
PSI	− 0.037 ± 0.011	− 0.042 ± 0.020	− 0.088 ± 0.177	0.035 ± 0.033^*^	0.001 ± 0.038	− 0.032 ± 0.016	0.002 ± 0.015	− 0.039 ± 0.033
SRmax [%/R-R]	6.12 ± 1.06	6.11 ± 0.64	6.24 ± 1.10	3.84 ± 0.55^*^	− 3.50 ± 0.43	− 3.40 ± 0.24	− 3.12 ± 0.31	− 2.03 ± 0.25^*^
SRe [%/R-R]	− 5.59 ± 1.03	− 6.39 ± 1.49	− 6.82 ± 1.52	− 5.50 ± 0.79	4.14 ± 0.87	3.97 ± 0.64	3.80 ± 0.52	3.42 ± 0.61
SRa [%/R-R]	− 3.00 ± 0.71	− 3.11 ± 0.65	− 3.35 ± 1.23	− 2.70 ± 1.22	1.90 ± 0.63	1.83 ± 0.35	1.86 ± 0.31	1.28 ± 0.63
SRe/a	1.95 ± 0.54	2.06 ± 0.25	2.17 ± 0.61	2.42 ± 1.10	2.48 ± 1.16	2.26 ± 0.67	2.11 ± 0.58	3.37 ± 1.84

The data are presented as the mean ± SD; *n* = 5–7, *P < 0.05 for Tgαq*44 mice vs. FVB mice at 4 months of age (Student’s *t* test or Mann–Whitney test). Legend: *m*, months; *Es*, systolic peak strain; *Ees*, end-systolic strain peak; *Epost*, post-systolic strain peak; *Tpeak*, time to end-systolic strain peak; *PSI*, post-systolic index; *SRmax*, strain rate maximum; *SRe*, early filling strain rate; *SRa*, atrial filling strain rate; *SRe/a*, early-to-atrial strain rate ratio; *R-R*, the time elapsed between two successive R-waves of the QRS signal on the electrocardiogram

### Perivascular cardiac fibrosis in HF in Tgαq*44 mice compared to age-matched FVB mice

**Fig. 2:**
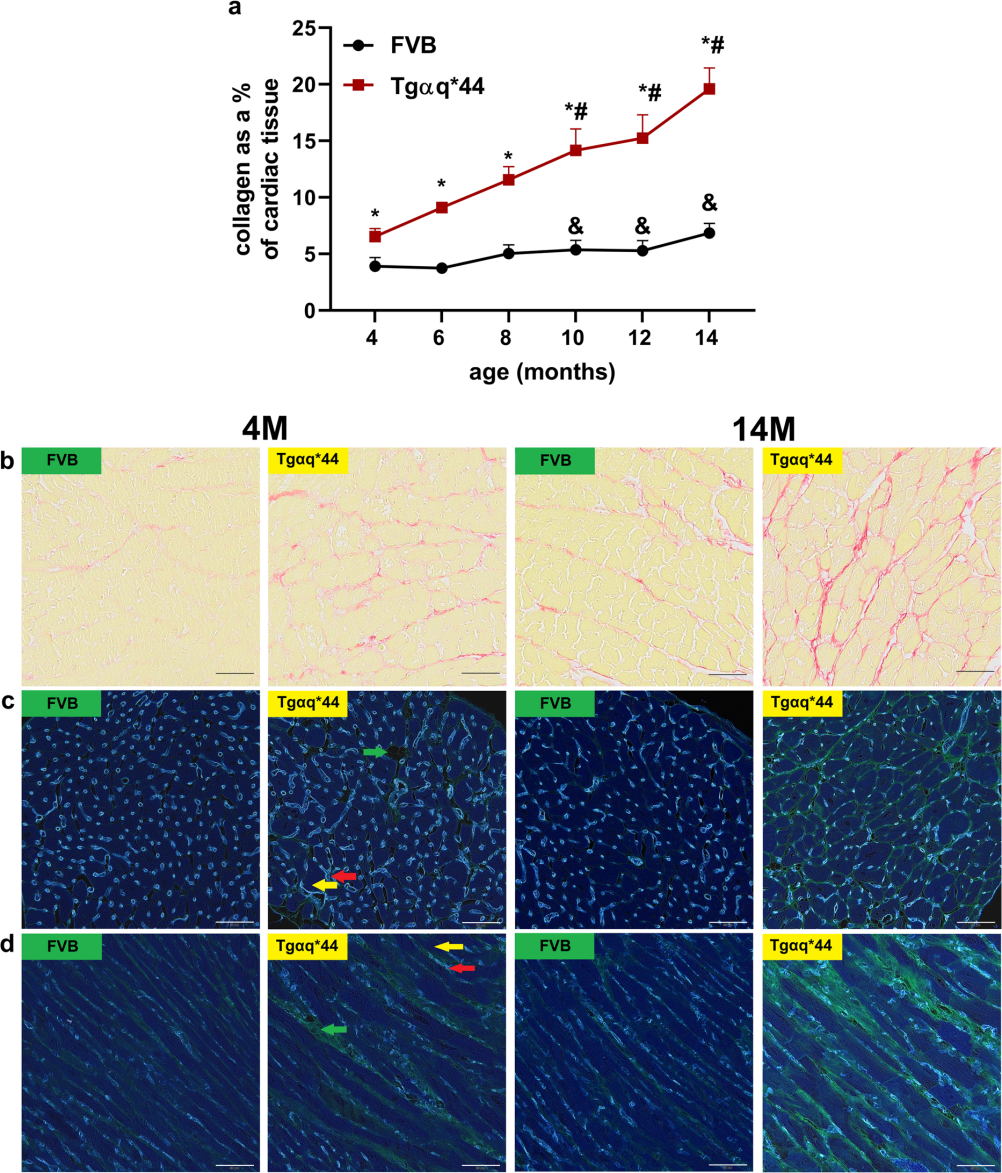
Progressive perivascular and interstitial fibrosis in the course of HF development in Tgαq*44 mice compared to age-related changes in FVB mice. Quantitative assessment of collagen deposition in cardiac tissue of Tgαq*44 mice and FVB mice, *n* = 6 (**a**). Representative images of collagen accumulation in cardiac tissue (**b**), transverse (**c**) and longitudinal sections (**d**) of cardiac tissue in 4- and 14-month-old Tgαq*44 mice and FVB mice showing colocalisation of capillaries and the extracellular matrix (scale bars indicate 50 µm – × 400 magnification). Collagen was stained with PSR; collagen fibres—red, cardiomyocytes—yellow (**b**). Cardiac tissue for visualisation of capillaries and the extracellular matrix was stained with lectin, PAS and picric acid and presented after colour inversion; capillaries—light blue area (red arrow); cardiomyocytes—navy blue area (yellow arrow), extracellular matrix – green area (green arrow)(**c, d**). The data are presented as the mean ± SD; *n* = 6, *P < 0.05 for Tgαq*44 mice vs. age-matched FVB mice (Student’s *t* test); ^#^*P* < 0.05 for older Tgαq*44 mice vs. 4-month-old Tgαq*44 mice; ^&^*P* < 0.05 for older FVB mice vs. 4-month-old FVB mice (one-way ANOVA with post hoc Tukey’s test or Kruskal–Wallis test with post hoc Dunn’s test). Legend: 4 M—4 months of age, 14 M—14 months of age

To test whether a progressive decline in basal CF in FVB and Tgαq*44 mice was associated with structural alterations in coronary microvasculature, cardiac collagen deposition was analysed by PSR staining, whereas the accumulation of extracellular matrix components and colocalisation with the network of blood vessels was visualised by staining with lectin, DAB as chromogen and PAS. As shown in Fig. 2, collagen content in cardiac tissue of 14-month-old FVB mice was increased compared to 4-month-old FVB mice (Fig. 2a, b), demonstrating age-dependent cardiac fibrosis. In 4-month-old Tgαq*44 mice, collagen deposition was already higher (6.54 ± 0.70%) compared with age-matched FVB mice (3.91 ± 0.78%) and similar to 14-month-old FVB mice (6.85 ± 0.84%, Fig. 2a, b). In 14-month-old Tgαq*44 mice, cardiac fibrosis was extensive (19.59 ± 1.87%, Fig. 2a, b). Similarly, cardiac extracellular matrix accumulation (lectin-based and PAS staining) displayed a modest increase in 14-month-old FVB mice compared to 4-month-old FVB mice but was more abundant in 4- and further increased in 14-month-old Tgαq*44 mice (Fig. 2c, d). As shown in Fig. 2, the accumulation of extracellular matrix components was localised around capillaries and cardiomyocytes as well as in the intercellular space.

### Alterations in coronary microvascular architecture in Tgαq*44 mice compared to age-matched FVB mice

**Fig. 3:**
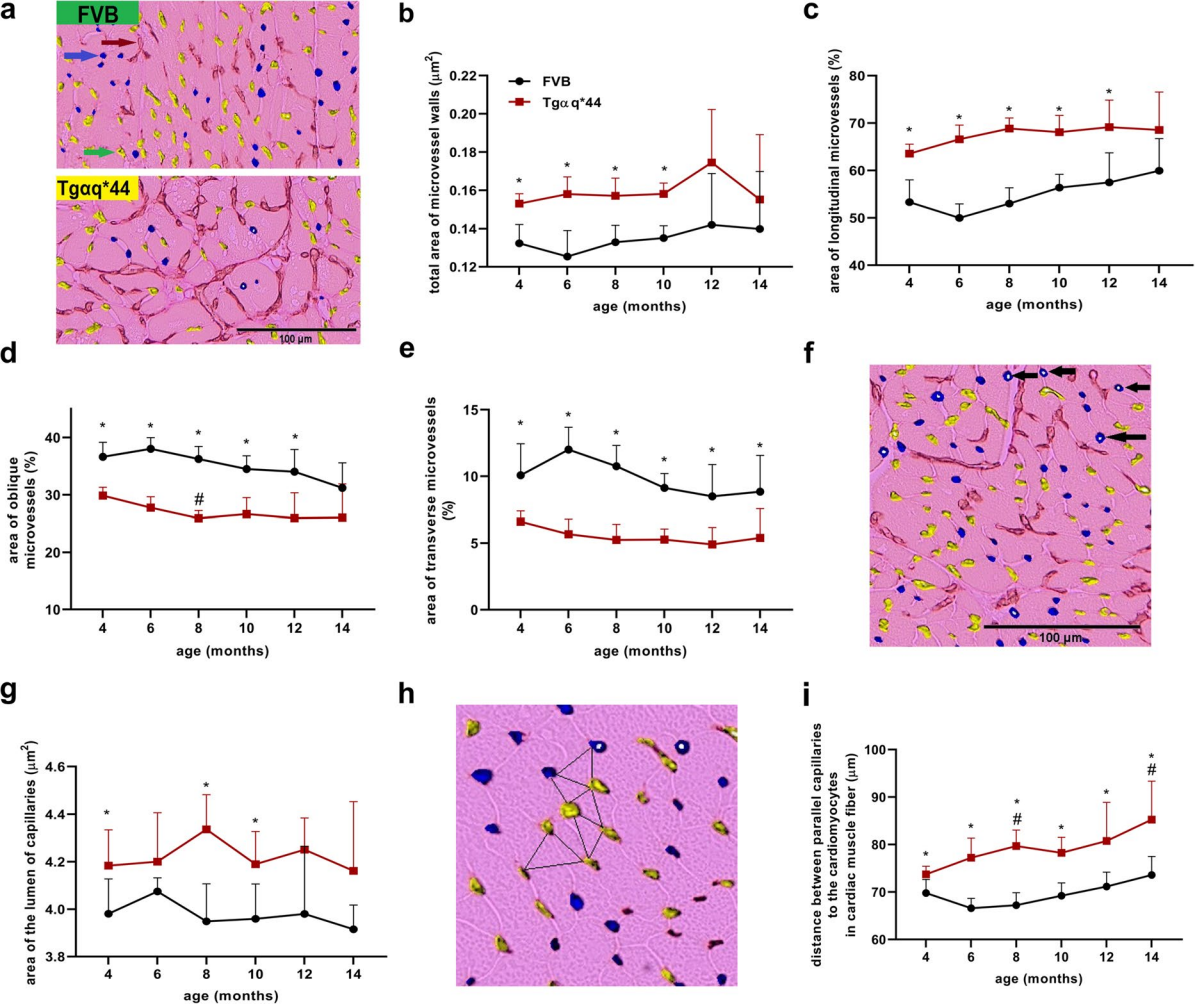
Alterations in cardiac capillary arrangement in the course of HF development in Tgαq*44 mice compared to age-related changes in FVB mice. Representative images of cross-sections of cardiac tissue of 14-month-old FVB and Tgαq*44 mice illustrating coronary capillary arrangement (scale bar indicates 100 µm – × 200 magnification) (**a**). Total area of microvessel walls (**b**) is presented in relation to area of cardiac tissue. Qualitative assessment of the area of longitudinal cardiac capillaries (**c**) representing capillaries arranged across the cardiomyocytes in the cardiac muscle fibre. Qualitative assessment of the area of oblique (**d**) and transverse cardiac capillaries (**e**) representing capillaries that go along cardiomyocytes in cardiac muscle fibre. Areas of longitudinal, oblique and transverse capillaries are presented as the percentage of the total area of microvessel walls that was normalised to the area of cardiac tissue. Images of cardiac tissue illustrating area of microvascular lumen (scale bar indicates 100 µm – × 200 magnification) (**f**) and the smallest averaged distances between cardiac capillaries (black triangles; close-up image from under × 200 magnification) (**h**). Area of the lumen of capillaries is presented as average area of vascular lumen of oblique and transverse cardiac capillaries that go along cardiomyocytes in cardiac muscle fibre (**g**). Distances between capillaries that go along cardiomyocytes in cardiac muscle fiber are presented as the smallest averaged distance between oblique and transverse cardiac capillaries (**i**). Cardiac tissue was stained with lectin and DAB. Images of cardiac tissue stainings by applying an algorithm; myocardium—pink area, area of longitudinal microvessels (brown arrow), area of oblique microvessels (green arrow), area of transverse microvessels (navy blue arrow), lumen of the microvessels (white area indicated by black arrows). The data are presented as the mean ± SD; *n* = 5–6, *P < 0.05 for Tgαq*44 mice vs. age-matched FVB mice (Student’s *t* test or Mann–Whitney test); ^#^*P* < 0.05 for older Tgαq*44 mice vs. 4-month-old Tgαq*44 mice (Kruskal–Wallis test with post hoc Dunn’s test)

To test whether increased interstitial and perivascular fibrosis in FVB and in Tgαq*44 hearts was associated with alterations in cardiac capillaries’ structural arrangement, a total area of capillaries and their organisation in relation to cardiomyocytes was analysed by lectin staining. In 14-month-old FVB mice, the cardiac microvasculature structure was fully preserved, as evidenced by lack of changes in several parameters measured: total area of microvasculature (Fig. 3a, b, S3), area of longitudinal microvessels (Fig. 3c), area of oblique microvessels (Fig. 3d), area of transverse microvessels (Fig. 3e), diameter of capillaries’ lumen (Fig. 3f, g) and density of capillaries running along cardiomyocytes (Fig. 3h, i). Surprisingly, in the hearts of Tgαq*44 mice, throughout the entire progression of HF, there were clear-cut alterations in the architecture of microvasculature: the total area of microvasculature, area of longitudinal vessels as well as the average area of the lumen of coronary microvessels were consistently higher (Fig. 3a, b, c, f, g, S3, S4, S5). In contrast, the area of oblique and transverse coronary microvessels were lower compared to age-matched FVB mice (Fig. 3d, e).

Importantly, the distances between capillaries adjacent to cardiomyocytes in the hearts of Tgαq*44 mice were significantly larger compared with respective distances between capillaries in the hearts from age-matched FVB mice (Fig. 3h, i).

These results extend earlier findings demonstrating cardiomyocyte hypertrophy in Tgαq*44 mice [53] and suggest a possible limitation in capillary coverage in Tgαq*44 mice hearts. Capillary architecture alterations in the Tgαq*44 mice were, however, not directly linked to macroscopic heart hypertrophy because the heart mass of Tgαq*44 mice compared to FVB mice increased only in older Tgαq*44 mice, and this change was caused mostly by a significant increase in atrial mass (Fig. S6).

### Ultrastructure of coronary microvasculature in Tgαq*44 mice compared to age-matched FVB mice

**Fig. 4:**
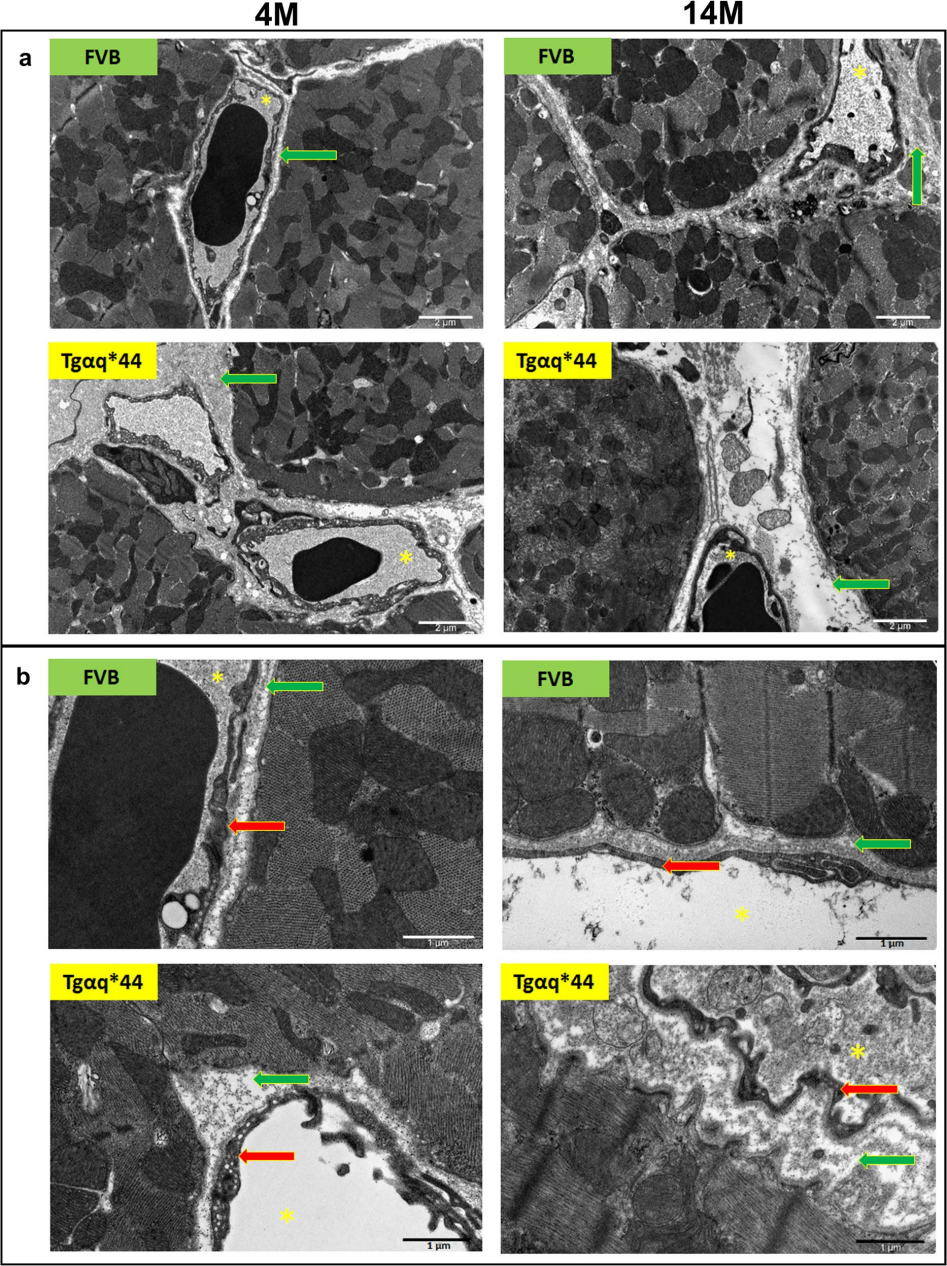
Ultrastructure of cardiac microvasculature in 4- and 14-month-old Tgαq*44 mice compared with age-related changes in FVB mice. TEM images of cross or longitudinal sections of cardiomyocytes and capillaries representing perivascular fibrosis (**a**) and ultrastructure of coronary endothelium (**b**) in the hearts of 4- and 14-month-old Tgαq*44 and age-matched FVB mice. Scale bars indicate 2 µm (**a**) and 1 µm (**b**). Legend: basal lamina of capillaries composed of extracellular matrix (green arrows), lumen of capillaries (yellow asterisks), endothelium (red arrows); 4 M—4 months of age, 14 M—14 months of age

To further define the structural nature of alterations in the coronary microvascular bed, TEM was used. The thickness of the basal lamina of coronary microvessels in 14-month-old FVB mice was slightly increased compared to 4-month-old FVB mice (Fig. 4a); however, the microvascular coronary endothelium ultrastructure was preserved (Fig. 4b). In the hearts of 4-month-old Tgαq*44 mice, the basal lamina of capillaries was thicker compared to age-matched FVB mice and further increased in the hearts of 14-month-old Tgαq*44 mice (Fig. 4a). The number of pinocytic vesicles in the endothelium of cardiac capillaries was increased in 4-month-old Tgαq*44 mice compared to age-matched FVB mice (Fig. 4b). Interestingly, in 14-month-old Tgαq*44 mice, the basal lamina around the cardiac capillaries was composed in large part of non-fibrotic, rather amorphous proteins (Fig. 4b).

### Alterations in cardiac and systemic activity of RAAS in Tgαq*44 mice compared to age-matched FVB mice

**Fig. 5:**
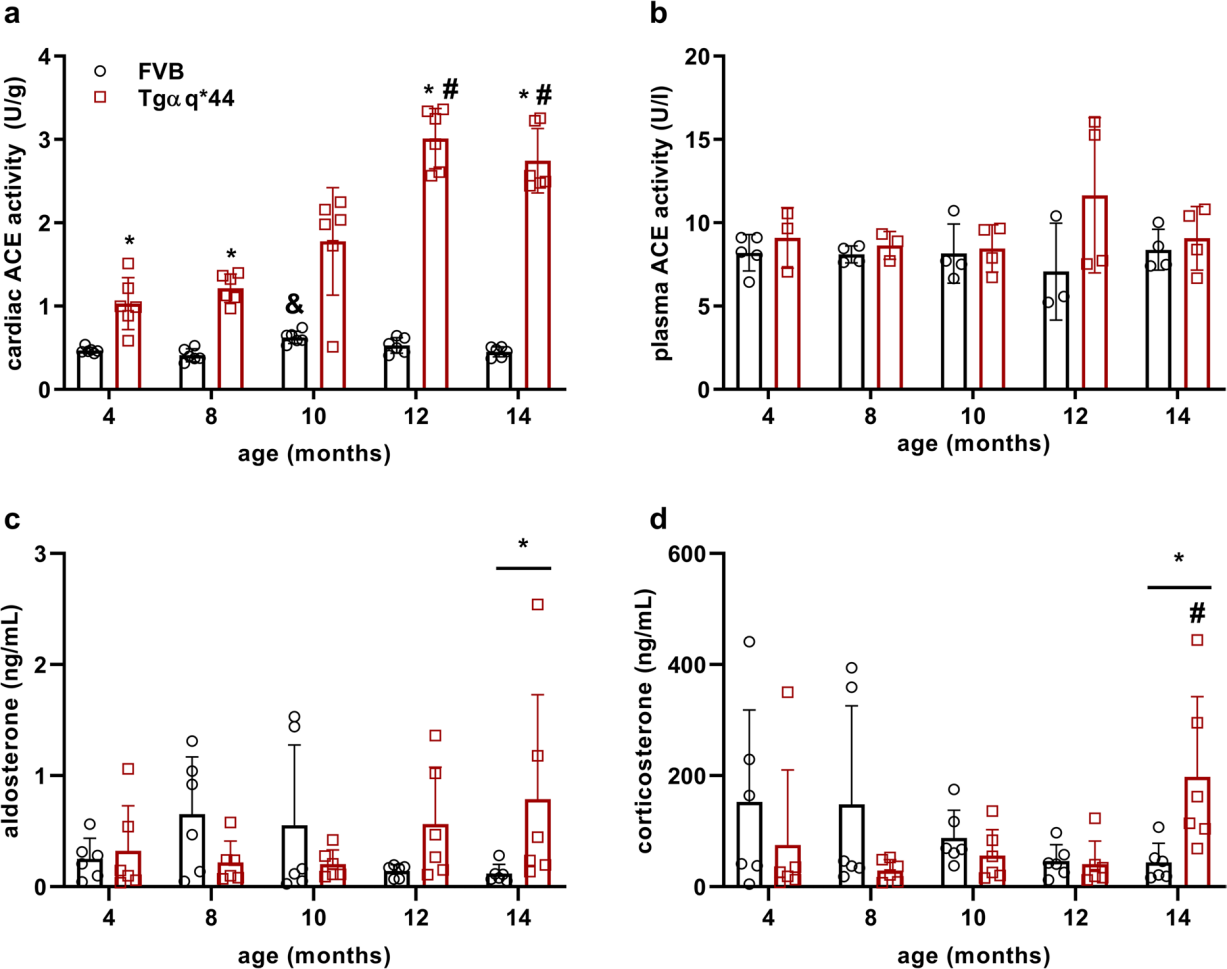
Progressive increase in cardiac ACE activity and mineralocorticoid plasma concentration in the course of HF development in Tgαq*44 mice compared to age-related changes in FVB mice. Cardiac ACE activity (**a**), plasma aldosterone (**c**) and corticosterone level (**d**) but not plasma ACE activity (**b**) were increased in the course of HF development in Tgαq*44 mice compared with ageing in FVB mice. The data are presented as the mean ± SD; *n* = 3–6, *P < 0.05 for Tgαq*44 mice vs. age-matched FVB mice (Student’s *t* test or Mann–Whitney test); ^#^*P* < 0.05 for older Tgαq*44 mice vs. 4-month-old Tgαq*44 mice; ^&^*P* < 0.05 for older FVB mice vs. 4-month-old FVB mice (one-way ANOVA with post hoc Tukey’s test or Kruskal–Wallis test with post hoc Dunn’s test)

To establish whether ageing in FVB mice and HF progression in Tgαq*44 mice are associated with cardiac and systemic angiotensin-aldosterone system activation, the activity of ACE in heart homogenates and in plasma, as well as the aldosterone concentration in plasma, were determined. ACE activity in the heart (Fig. 5a) and plasma (Fig. 5b), as well as plasma aldosterone concentration, were not altered in 14-month-old FVB mice compared to 4-month-old FVB mice (Fig. 5c). In contrast, in 4-month-old Tgαq*44 mice, cardiac ACE activity was higher compared to age-matched FVB mice and progressively increased in 8-, 10-, 12- and 14-month-old Tgαq*44 mice (Fig. 5 a). In contrast, plasma ACE activity remained almost unchanged in all age groups of Tgαq*44 mice compared to age-matched FVB mice (Fig. 5b). However, the aldosterone level in the plasma was higher in 14-month-old Tgαq*44 mice compared to age-matched FVB mice (Fig. 5c) and was paralleled by increased plasma corticosterone concentration (Fig. 5d) without significant alterations in progesterone or testosterone levels (Fig. S1).

There were some significant alterations in blood cell count in Tgαq*44 mice compared with age-matched FVB mice, with a decreased relative number of lymphocytes in 4- and 14-month-old Tgαq*44 mice compared to age-matched FVB mice as well as in 14-month-old FVB mice when compared to 4-month-old FVB mice (Supplementary Table S2).

### Changes in cardiac transcriptome along cardiac ageing process in FVB mice

**Fig. 6:**
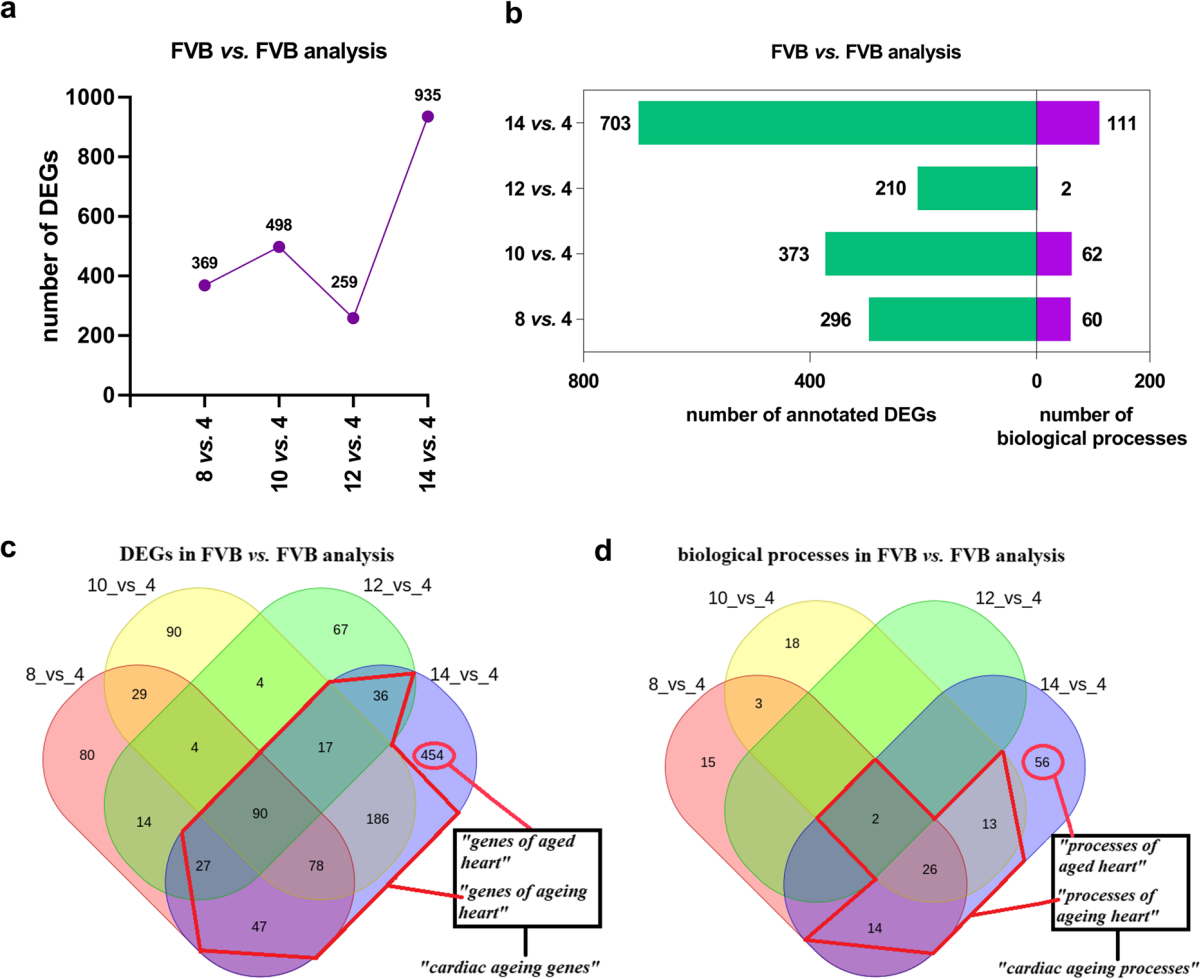
Overview of DEGs and over-represented biological processes in FVB vs. FVB analysis. Number of DEGs (**a**) as well as number of annotated DEGs to the respective number of over-represented biological processes in FVB vs. FVB analysis (**b**). Venn diagrams presenting common and unique DEGs (**c**) and over-represented biological processes (**d**) in FVB vs. FVB analysis in all age groups. In light red circles, there are ‘*genes of aged heart*’ (**c**) and ‘*processes of aged heart*’ (**d**) that are represented by DEGs and over-represented biological processes, which occurred only in 14-month-old FVB mice (but not in younger age groups) in FVB vs. FVB analysis. In red polygons, there are ‘*genes of ageing heart*’ (**c**) and ‘*processes of ageing heart*’ (**d**) that are represented by DEGs and over-represented processes, which occurred in 14-month-old FVB mice as well as at least in one of the younger FVB mice age groups in FVB vs. FVB analysis (Supplementary Table S7). Legend: ‘*cardiac ageing genes*’ represent ‘*genes of aged heart*’ as well ‘*genes of ageing heart*’; ‘*cardiac ageing processes*’ represent ‘*processes of aged heart*’ as well ‘*processes of ageing heart*’

To determine cardiac transcriptome age-dependent changes in FVB mice, the numbers of cardiac DEGs and over-represented biological processes in FVB mice during ageing were evaluated (Supplementary Table S3, S4, S5). The highest number of ageing-dependent DEGs (935) and over-represented biological processes (111) in the cardiac transcriptome of FVB mice was observed in 14-month-old FVB mice (Fig. 6a, b). Interestingly, there were 454 DEGs and 56 over-represented biological processes present only in the 14-month-old group (but not in younger age groups) in FVB vs. FVB analysis (Fig. 6c, d, Supplementary Table S6).

### Changes in cardiac transcriptome along HF progression in Tgαq*44 mice in relation to age-dependent transcriptomic changes in FVB hearts

**Fig. 7:**
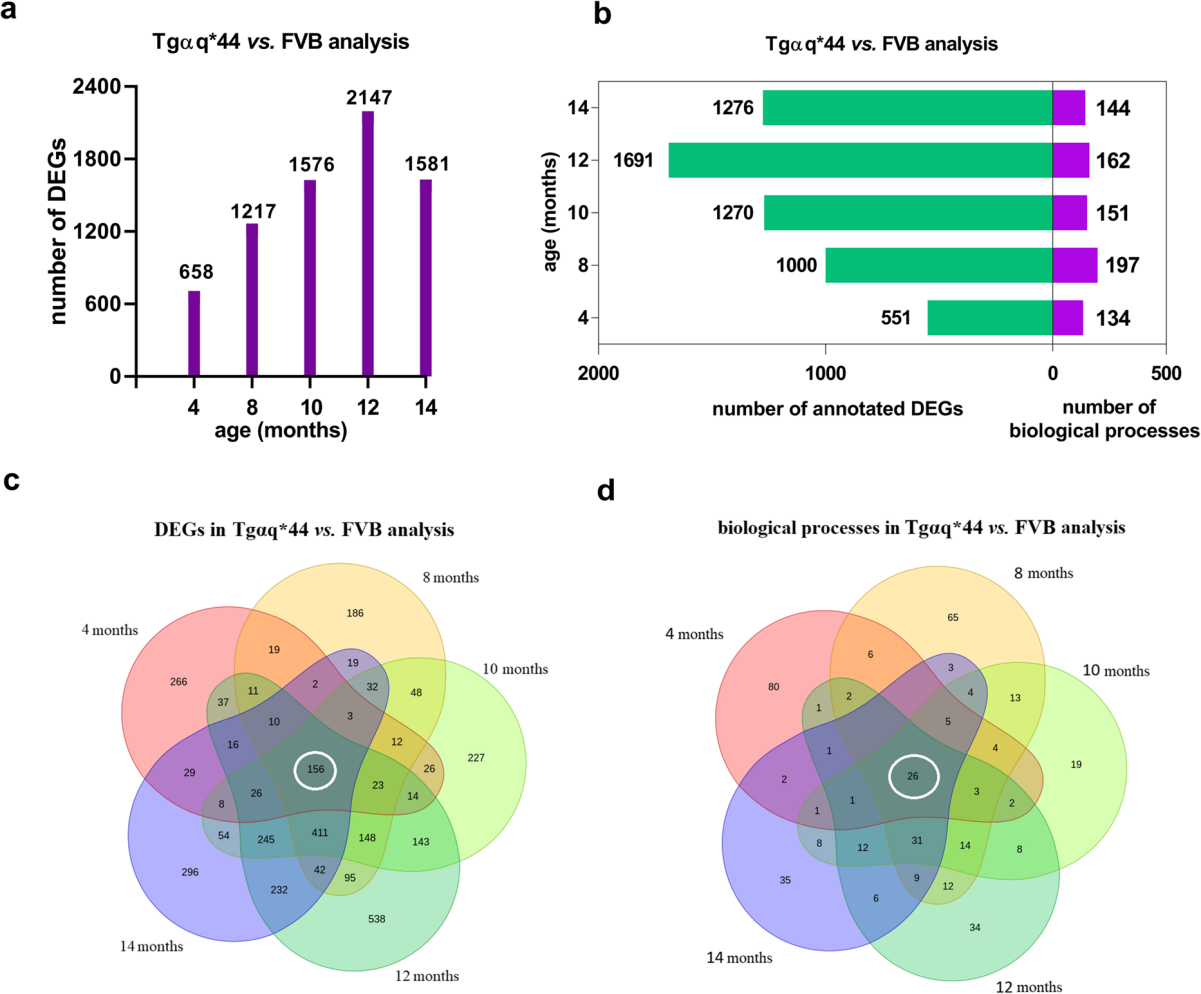
Overview of DEGs and over-represented biological processes in Tgαq*44 vs. FVB analysis. Number of DEGs (**a**) as well as number of annotated DEGs to the respective number of over-represented biological processes in Tgαq*44 vs. FVB analysis (**b**). Venn diagrams presenting common and unique DEGs (**c**) and over-represented biological processes (**d**) in Tgαq*44 vs. FVB analysis in all age groups. In white circles, there are DEGs (**c**) and over-represented biological processes (**d**) that occurred in Tgαq*44 vs. FVB analysis in all age groups

In Tgαq*44 vs. FVB analysis, the number of DEGs and over-represented biological processes displayed a distinct pattern as observed in FVB vs. FVB analysis (Fig. 7a, b, Supplementary Table S8, S9, S10) with a maximal number of DEGs in 12-month-old mice (2147), whereas over-represented biological processes stayed in the similar range in 4- to 14-month-old mice (134–162) with a markedly higher number in 8-month-old mice (197). Of note, there were 156 DEGs (Fig. 7c, Supplementary Table S11) and 26 over-represented biological processes (Fig. 7d, S7) present in all age groups in comparisons between Tgαq*44 and age-matched FVB mice.

**Fig. 8:**
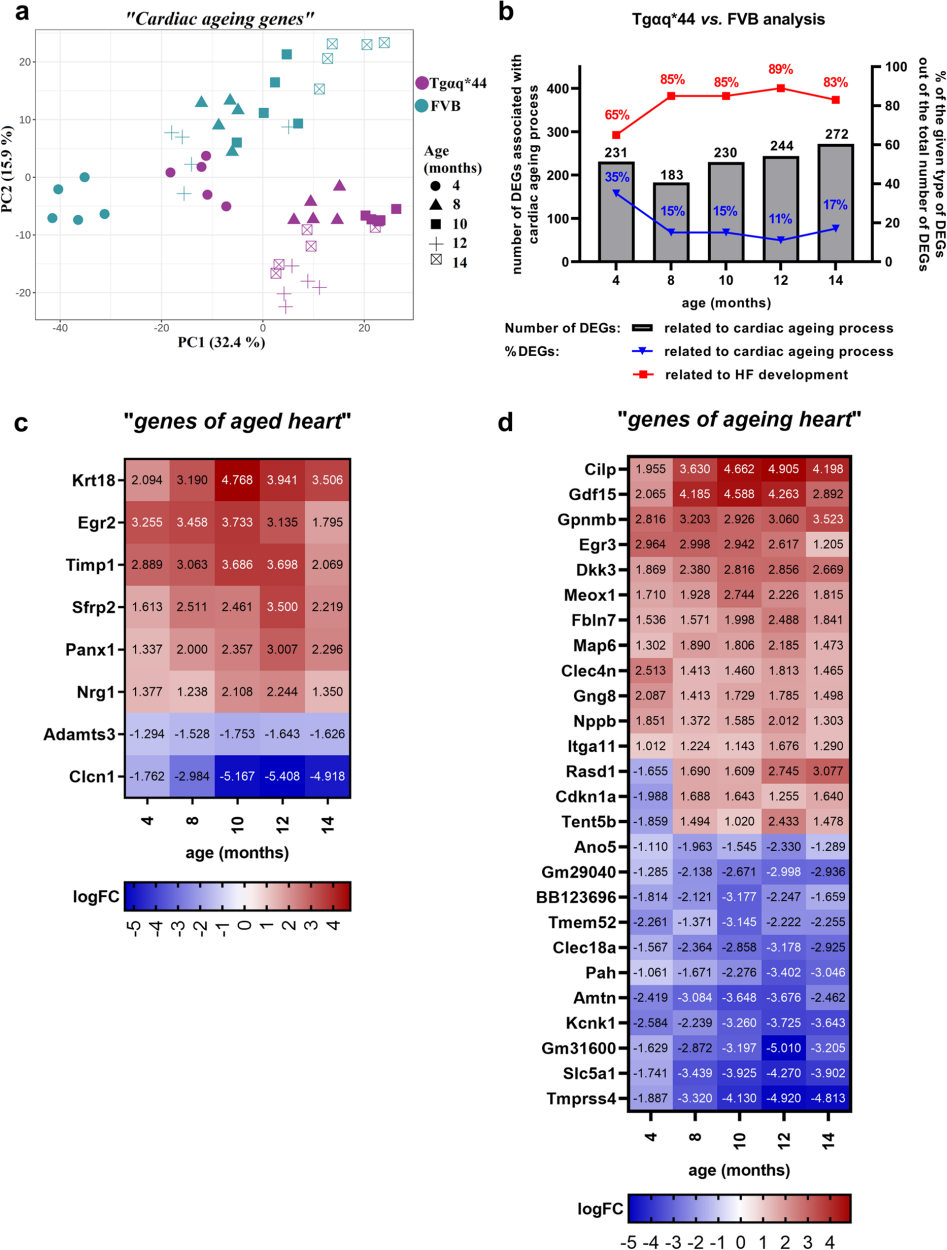
‘*Cardiac ageing genes*’ in Tgαq*44 mice vs. FVB mice in a course of HF development. PCA presenting gene expression variances of ‘*cardiac ageing genes*’ (935 genes that involve 454 ‘*genes of aged heart*’ and 481 ‘*genes of ageing heart*’) in Tgαq*44 and FVB hearts at the age of 4–14 months (**a**). Contribution of differentially expressed ‘*cardiac ageing genes*’ in all differentially expressed genes in Tgαq*44 vs. FVB analysis (**b**). Heatmaps of 8 ‘*genes of aged heart*’ (**c**) and 26 ‘*genes of ageing heart*’ (**d**) that were differentially expressed in Tgαq*44 vs. FVB analysis in all age groups representing genes characteristic for early-activated and long-lasting ageing process in the cardiac transcriptome of Tgαq*44 mice. Legend: HF – heart failure

To identify whether cardiac ageing in Tgαq*44 mice is altered compared to FVB mice, ‘*cardiac ageing genes*’ (Fig. 6c) were plotted in a PCA analysis. As shown in Fig. 8a, the pattern of changes in gene expression variances of ‘*cardiac ageing genes*’ in FVB and Tgαq*44 mice was different. ‘*Cardiac ageing genes*’ in 4- to 14-month-old FVB mice were wider spread than in the respective comparison for Tgαq*44 mice. Furthermore, gene expression variances for ‘*cardiac ageing genes*’ for 4-month-old Tgαq*44 mice stayed close to those of 8-month-old FVB mice, whereas those for 8- to 14-month-old Tgαq*44 mice were separated and appeared to be grouped together (Fig. 8a).

Interestingly, ‘*cardiac ageing genes*’ accounted for as much as 35% of all DEGs found in Tgαq*44 vs. FVB analysis at the age of 4 months and amounted to approximately 15% of all DEGs in Tgαq*44 vs. FVB analysis at 8–14 months of age (Fig. 8b). Indeed, ‘*cardiac ageing genes’* were well represented in DEGs for 4- to 14-month-old Tgαq*44 mice vs. age-matched FVB mice, and their number ranged between 183 and 272 genes (Fig. 8b, Supplementary Table S12).

Furthermore, among 156 genes that occurred as DEGs in Tgαq*44 vs. FVB analysis along with the entire development of HF (Fig. 7c, Supplementary Table S11), there were as many as 34 ‘*cardiac ageing genes*’ (8 ‘*genes of aged heart*’ and 26 ‘*genes of ageing heart*’ (Fig. 8c, d, Supplementary Table S13, S14)), pointing out that these ‘*cardiac ageing genes*’ were early activated, and their activation was sustained throughout HF development in Tgαq*44 mice. This group of genes included *Gdf15*, *Cdkn1a* (involved in cellular senescence, and in the case of *Gdf15* also in the fibrotic process) [54–56], *Egr2* [57], *Egr3* [58], *Timp1* [59], *Sfrp2* [60], *Itga11* [61] (involved in fibrosis) and *Cilp* (involved in the cardiac anti-fibrotic process by inhibiting TGF-β signalling) [62].

**Fig. 9:**
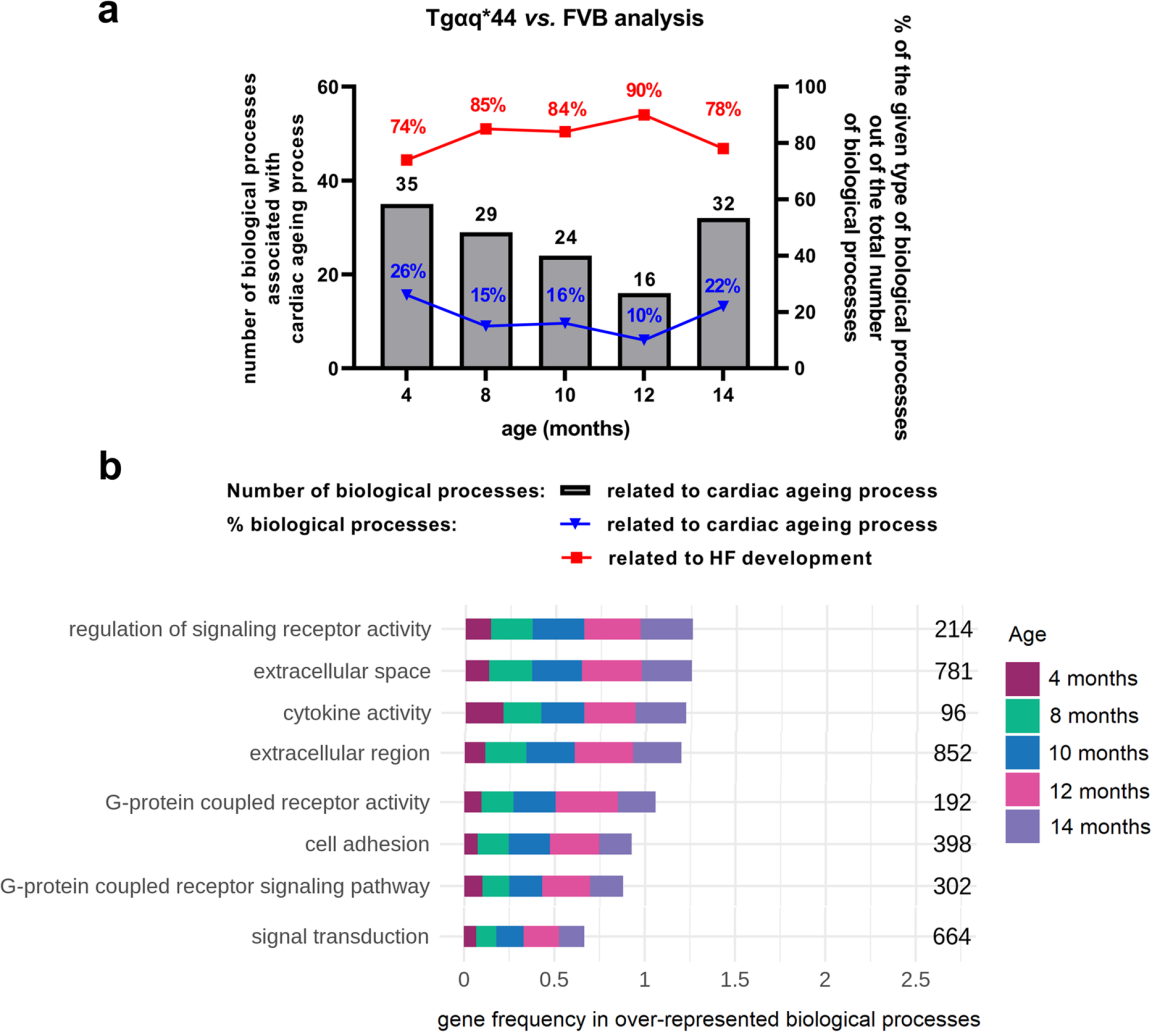
‘*Cardiac ageing processes*’ in Tgαq*44 mice vs. FVB mice in a course of HF development. Contribution of ‘*cardiac ageing processes*’ in all over-represented biological processes in Tgαq*44 vs. FVB analysis (**a**). Gene frequency in selected ‘*processes of ageing heart*’ (**b**) that were initiated in all Tgαq*44 mice age groups (in Tgαq*44 vs. FVB analysis) representing biological processes characteristic for early-activated and long-lasting ageing process in the cardiac transcriptome of Tgαq*44 mice. Legend: HF – heart failure; numbers shown (**b**) represent number of genes included in respective biological processes

The ‘*cardiac ageing processes*’ identified in FVB mice (Fig. 6d) were also well represented in young Tgαq*44 mice and accounted for 26% of over-represented biological processes in 4-month-old Tgαq*44 mice vs. age-matched FVB mice (Fig. 9a). The contribution of ‘*cardiac ageing processes*’ in Tgαq*44 vs. FVB analysis in other age groups was in the range of 10–22% and included 16–32 over-represented biological processes (Fig. 9a, Supplementary Table S12). Furthermore, there were 8 ‘*processes of ageing heart*’ that were over-represented in all experimental groups between 4 and 14 months of age in Tgαq*44 vs. FVB analysis, pointing out their early and sustained activation in the hearts of Tgαq*44 mice (Figs. 9b, 7d). Although the gene frequency in these selected ‘*processes of ageing heart*’ was the most abundant in 12-month-old Tgαq*44 mice in Tgαq*44 vs. FVB analysis, this frequency was quite similar in 8- to 10- and 14-month-old Tgαq*44 mice, underscoring the persistent presence of cardiac ageing-related biological processes in Tgαq*44 mice during HF development (Fig. 9b).

## Discussion

In the present work, we comprehensively characterised the progression of HF in Tgαq*44 mice on functional, morphological and transcriptomic levels in comparison to age-matched FVB mice with the aim of identifying the elements of chronic HF pathophysiology that could be related to cardiac ageing. We provide evidence that the cardiac transcriptome representative of cardiac ageing as identified in FVB mice (‘*cardiac ageing genes*’, ‘*cardiac ageing processes*’) was in part recapitulated in Tgαq*44 mice and contributed to as much as 35% of DEGs and 26% of over-represented biological processes in the hearts of young 4-month-old Tgαq*44 mice if compared to age-matched FVB mice and was also well represented in older Tgαq*44 vs. age-matched FVB mice. Furthermore, among common DEGs and over-represented biological processes present in all age groups in the analysis of Tgαq*44 vs. age-matched FVB mice, there were 34 ‘*cardiac ageing genes*’ (in the group of 156) and 8 ‘*cardiac ageing processes*’ (in the group of 26). This finding was also supported by PCA analysis of ‘*cardiac ageing genes*’ expression variances, providing unprecedented evidence of accelerated and persistent cardiac ageing in chronic HF in Tgαq*44 mice.

Accordingly, our results show that cardiac transcriptomic changes compatible with cardiac ageing represent an important contribution to the cardiac transcriptome of hearts taken from Tgαq*44 mice not only at the end-stage HF at the age of 12–14 months that could clearly show signs of cardiac ageing even under physiological conditions but also at earlier phases of HF progression in this model in younger Tgαq*44 mice at the age that is not physiologically associated with cardiac ageing.

**Fig. 10:**
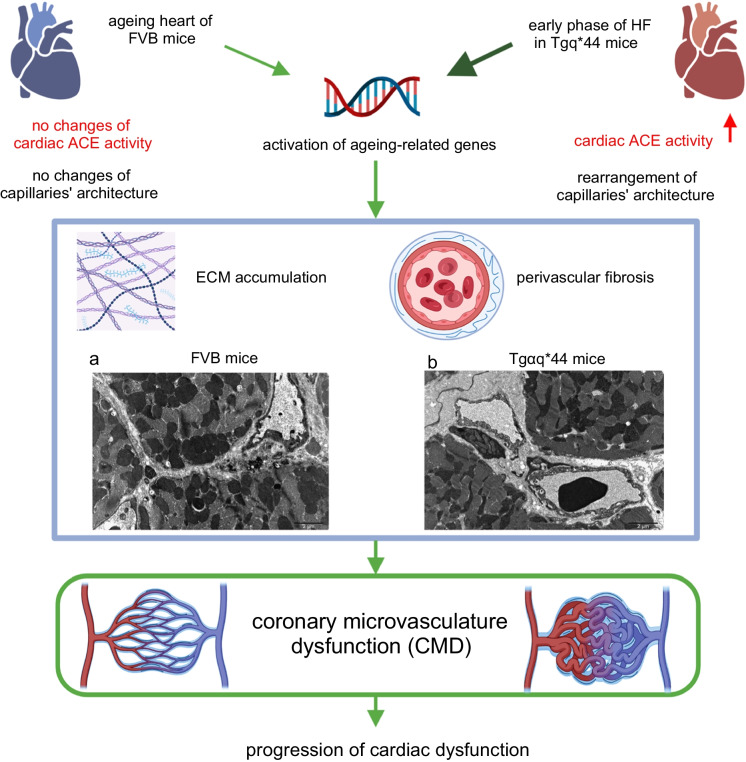
CMD in pathophysiology of HF in Tgαq*44 mice and in ageing-related cardiac dysfunction in old FVB mice. Activation of ageing-related genes in the hearts of Tgαq*44 mice at early phase of HF and in the hearts of old FVB mice trigger increased cardiac extracellular matrix (ECM) accumulation and perivascular fibrosis leading to CMD and cardiac dysfunction. **a**, **b** show ultrastructure of cardiac microvasculature in 14-month-old FVB mice and 4-month-old Tgαq*44 mice, respectively. Tgαq*44 mice but not old FVB mice display increase in cardiac ACE activity and rearrangement of capillaries’ architecture. Yet, CMD is shared by Tgαq*44 mice and old FVB mice and represent an intersection of cardiac ageing and HF pathophysiology. An image created with BioRender.com

Importantly, among the ‘*cardiac ageing genes*’, many of them were related to extracellular matrix remodelling and fibrosis, compatible with the structural impairment of coronary microvasculature shown here. In fact, the cardiac ageing phenotype in FVB mice included impairment of basal CF with extracellular matrix remodelling and perivascular and interstitial fibrosis. In Tgαq*44 mice, basal CF was more severely impaired and was associated with distinct rearrangement of the capillary architecture and substantially more pronounced extracellular matrix remodelling and perivascular and interstitial fibrosis (Fig. 10).

Altogether, our work reveals that chronic HF in Tgαq*44 mice is associated with accelerated and persistent cardiac ageing exemplified by prominent extracellular matrix remodelling and perivascular fibrosis in coronary microcirculation resulting in coronary microvascular dysfunction (CMD) (Fig. 10).

Here, we took advantage of a unique murine model of slowly developing HF. In Tgαq*44 mice, initially developed by Mende et al. [40], cardiomyocyte-specific overexpression of the constitutively active Gαq protein imitates excessive neurohormonal drive in the heart and results in slowly progressing cardiac pathology that mimics HF in humans on molecular, morphological, phenotypic and functional levels [40, 42, 53, 63–67]. Importantly, in Tgαq*44 mice, the progression of HF is prolonged with distinct early (at the age of 4–6 months), transition (at 8–10 months of age) and end-stage phases of cardiac impairment (at the age of 12–14 months) [41, 42]. Previous studies using Tgαq*44 mice extensively characterised HF pathophysiology and revealed the complex phenotype of the disease in this model. HF development in Tgαq*44 mice is associated with the foetal phenotype, including increased β-myosin heavy chain and atrial natriuretic peptide (ANP) mRNA expression [40], alterations in ACE-2/angiotensin 1–7 and ACE/angiotensin II pathways [42], cardiac mitochondrial dysfunction and altered cardiac metabolism [53, 65], pathological cardiac tissue reorganisation with altered desmin expression [66], fibrosis [66], coronary endothelial dysfunction [63], cardiac oxidative stress [68], systemic endothelial dysfunction associated with erythropathy [69] and brain endothelial dysfunction with cognitive impairment [70]. Tgαq*44 mice also display impaired exercise capacity [71, 72] and right ventricular HF with congestive hepatopathy (unpublished). In the present work, we extended the existing knowledge on the pathophysiology of HF in Tgαq*44 mice by demonstrating the contribution of the cardiac ageing phenotype to HF pathophysiology, using age-matched FVB wild-type mice as a reference model to define the cardiac ageing phenotype. We provided evidence for accelerated cardiac ageing in Tgαq*44 mice.

We demonstrated that cardiac ageing in 14- to 16-month-old FVB mice involves mild perivascular fibrosis of coronary microcirculation, impairment in basal CF and altered diastolic function. Tgαq*44 mice displayed robust perivascular fibrosis in coronary microcirculation with extracellular matrix deposition suggesting functional impairment and development of CMD at the transition and end-stage HF, associated with pronounced impairment of diastolic and systolic cardiac function. Altogether, based on our functional, morphological and transcriptomic results, we claimed that the coronary microvasculature represented a *locus minoris resistentiae* of the accelerated cardiac ageing in HF.

Impairment of basal CF peak velocity in cardiac ageing (as observed in 16-month-old FVB mice) was associated with a decrease in the left ventricular FR, most likely linked to fibrosis and increased cardiac stiffness (observed as decreased E/A ratio) [73–75] that however did not result in impaired contractility (preserved EF, SV, ESV as well as circumferential/ radial strains and strain rates). In contrast, in young 4-month-old Tgαq*44 mice, left ventricular strains (both: circumferential and radial) were already impaired as well as the key cardiac functional parameters (EF, CO, SV), confirming impaired contractility along with reduced diastolic function, whereas impaired basal CF peak velocity was present at the transition phase of HF in 8-month-old Tgαq*44 mice. Of note, IVCT—a known prognostic parameter in cardiac diseases [76, 77]—was prolonged, resulting in the shortening of FT and subsequently leading to increased rather than decreased FR, as observed in FVB mice, indicating that the mechanisms of cardiac dysfunction in HF in Tgαq*44 mice and in cardiac ageing in 14- to 16-month-old FVB mice are mechanistically distinct. Indeed, our results suggest that mechanisms of cardiac dysfunction in Tgαq*44 and FVB mice might be related and not related to cardiac ACE and systemic activation of RAAS pathways, respectively. Despite differences in cardiac endotype in old FVB compared to Tgαq*44 mice, alterations in microvasculature were shared by Tgαq*44 mice and old FVB mice and represent an intersection of cardiac ageing and HF pathophysiology. These results agree with a clinical study demonstrating that depressed resting myocardial blood flow was a predictor of death or progression in idiopathic left ventricular dysfunction [78]. Furthermore, a recent meta-analysis provided overwhelming evidence that CMD assessed as impaired CF reserve was strongly associated with an increased risk of all-cause mortality [79], further underscoring the pathophysiological importance of CMD in cardiac diseases, including HF.

In the present work, we demonstrated evident perivascular fibrosis in coronary microcirculation that could determine malfunction of the coronary microvasculature in Tgαq*44 mice. In the hearts of 4-month-old Tgαq*44 mice, perivascular fibrosis was quantitatively similar to that observed in hearts taken from 14-month-old FVB mice and then considerably increased along HF progression in Tgαq*44 mice. Thickening of basal lamina and accumulation of extracellular matrix components around cardiac capillaries composed of non-fibrotic, amorphous proteins were prominent features of structural alterations detected in Tgαq*44 hearts. Such structural modifications are similar to age-related changes that were described not only in coronary but also in other vascular beds and are known to result in age-dependent blood flow decline in many organs [19]. In the present work, we did not assess the precise composition of the extracellular matrix around ageing cardiac capillaries, but this matrix could comprise increased levels of glycoproteins, integrins and matricellular proteins, as previously shown in the extracellular matrix of ageing hearts [80]. Interestingly, in patients with non-ischemic HF, coronary perivascular fibrosis was the major determinant of the impairment of coronary blood flow compared with interstitial fibrosis or cardiac function [81], further supporting our conclusion that perivascular fibrosis is an important determinant of CF and an important player in CMD in Tgαq*44 hearts. Impaired NO-dependent function and increased oxidative stress could also contribute to CMD [77, 82] in this model but most likely more so in later stages because NO-dependent vasodilation was impaired in 14-month-old Tgαq*44 mice [63].

It was quite a puzzling finding that in Tgαq*44 mice but not in FVB mice displayed such a profound rearrangement of cardiac capillary architecture. The total coronary vasculature volume was increased, as evidenced by an increased total area of microvasculature associated with a notable increase in area of longitudinal capillaries and a relative fall in the number of transverse capillaries in relation to size of hypertrophic cardiomyocytes. Importantly, capillaries lost their linear orientation and consistent shape but exhibited highly branched irregular, tortuous arrangements. Quite interestingly, similar alterations in coronary microvasculature were described in rats after aortic banding and ischaemia reperfusion with subsequent aortic debanding [83]. The authors suggested that significant structural abnormalities of coronary capillaries may drastically stagnate haemodynamics in the myocardium and increase resistance to blood flow and contribute to HF in this model in particular; these changes might be linked to narrow capillary branches that bridge larger capillaries [83]. Furthermore, the capillaries’ tortuosity could result in a reduction in shear stress [83] and vasodilatory malfunction [84]. In our hands, lumen of capillaries increased, suggesting that their highly branched tortuous arrangements are mainly responsible for limitation for myocardial flow.

Our findings of the profound response of cardiac capillaries’ architecture to HF progression in Tgαq*44 mice agree with reports showing that cardiac hypertrophy was linked to the initial increase in capillary density that was, however, later reduced [85]. Indeed, functional coronary responses in the isolated heart seemed to be higher in 4-month-old Tgαq*44 mice compared to age-matched FVB mice. Furthermore, in 8-month-old and older Tgαq*44 mice there was a progressive impairment of basal CF measured in vivo paralleled with the impairment of cardiac function that was significant and more severe compared with 4-month-old Tgαq*44 mice [41, 42], involving systolic and diastolic impairment with a prominent increase in FR (which is a hallmark of restrictive diastolic impairment) [86, 87] and progressive exercise capacity impairment [72]. Finally, the end-stage phenotype was observed in 12- to 14-month-old Tgαq*44 mice [41, 42] that could be partially linked to the progressive CMD.

It is important to note that the decreased basal CF peak velocity observed in vivo was not confirmed in the isolated murine heart retrogradely perfused with electrolyte solution, pointing out that some important pathophysiological elements affecting basal CF regulation in vivo cannot be fully recapitulated in the isolated heart. Obviously, isolated heart preparations were used previously to detect alterations in coronary reactivity and revealed changes in NO- and prostacyclin-dependent coronary vascular function in various murine models [88–92], including Tgαq*44 mice [63]. However, the impairment of coronary reserve and bradykinin-dependent responses in Tgαq*44 mice, detected in the isolated murine heart, was a relatively late phenomenon occurring in 14-month-old Tgαq*44 mice [63], whereas in 8-month-old Tgαq*44 mice as shown here, coronary reserve and bradykinin-induced vasodilation in the isolated heart was fully preserved in contrast to deteriorated basal CF in vivo*.* It seemed that preserved NO-dependent dilative properties of coronary arteries in 8-month-old Tgαq*44 mice in the isolated perfused heart in Langendorff mode might be due to non-physiologically dilated coronary vessels in perfusion Krebs–Henseleit buffer of low oxygen carrying capacity [93] and not optimally stimulated coronary vessels by shear stress the most powerful physiological regulator of NO production via endothelial nitric oxide synthase [94]. Perhaps using isolated working heart would be a better approach for detecting basal CF impairment, contrasting with the Langendorff method used because in the working heart model the coronary perfusion is linked to the heart workload [95].

It is important to add that we did not measure directly coronary microvascular function in the present work in Tgαq*44 or in FVB mice in vivo to confirm CMD. Invasive measurements are increasingly performed in humans to diagnose CMD. However available methods to evaluate microcirculation function in humans or in bigger animals [30, 96, 97] cannot be applied in murine heart due to its small size and rapid pacing and other emerging techniques for direct CMD analysis were not available in our studies [98].

To supplement studies characterising coronary microvasculature in FVB and Tgαq*44 mice, we performed a comprehensive transcriptomic analysis to provide evidence that robust perivascular fibrosis and extracellular matrix remodelling in coronary microcirculation in Tgαq*44 mice may be partially linked to accelerated cardiac ageing (Fig. 10). We defined a group of genes and biological processes characteristic of cardiac ageing in wild-type FVB mice and, to our surprise, identified that approximately 15% of all DEGs in Tgαq*44 vs. FVB analysis at the age of 8–14 months represented cardiac ageing genes, with as much as 35% of cardiac ageing genes found at the age of 4 months in Tgαq*44 vs. FVB analysis. These results provided unprecedented evidence for an early and sustained contribution of cardiac ageing to HF pathophysiology. Interestingly, 34 ‘*cardiac ageing genes*’ were differently expressed in the cardiac transcriptome in Tgαq*44 mice (if compared to age-matched FVB mice) and stayed differently expressed along the entire HF development, underscoring their relevance to cardiac ageing-related contribution to pathophysiology from the early to the late phases of HF. Among these genes, we identified the early and sustained activation of genes’ expression related to senescence (*Cdkn1a*, *Gdf15*) [54, 55], fibrosis (*Timp1*, *Egr2*, *Egr3*, *Gdf15*, *Sfrp2*, *Itga11*) [56–61] and extracellular matrix remodelling (*Timp1*, *Gpnmb*) [82, 99]. These processes were all characteristic of ageing hearts [80, 100, 101] and ageing vasculature [102, 103]. Indeed, 8 ‘*cardiac ageing processes*’ included ‘cytokine activity’, ‘cell adhesion’, ‘extracellular region’ and ‘extracellular space’, underscoring the importance of inflammation, extracellular matrix remodelling and fibrosis that was again compatible with the postulated mechanisms of perivascular remodelling and fibrosis of coronary microcirculation (Fig. 10) [6, 104].

Interestingly, in the group of selected 34 ‘*cardiac ageing genes*’, there were genes that could be assigned to cardiac adaptation (*Nrg1*, *Cilp*, *Nppb*) [62, 105, 106] or maladaptive processes (*Cdkn1a*, *Timp1*, *Egr2*, *Egr3*, *Gdf15*, *Sfrp2*, *Itga11*) [54–61], underscoring the importance of crosstalk between mechanism activating and inhibiting HF development among those activated by accelerated cardiac ageing in HF.

It is noteworthy that the mechanisms previously reported to be involved in increased capillary density in cardiac hypertrophy were not altered in the cardiac transcriptome in Tgαq*44 mice (vascular endothelial growth factor (VEGF)- and hypoxia-inducible factor-1 (Hif-1)-dependent pathways) [85, 107]. On the other hand, neuregulin 1 (NRG1) [108] was upregulated throughout HF development. Here, we did not decipher the mechanisms involved in cardiac capillaries’ response to HF progression. Most likely, there are multiple compensative mechanisms that could regulate adaptive coronary microvascular changes that become maladaptive or are negatively regulated in Tgαq*44 mice along the progression of HF. For example, in our previous studies, we demonstrated alterations in ACE-2/angiotensin 1–7- and epoxyeicosatrienoic acid (EET)-dependent pathways [42, 109] but many other pathways may be involved in adaptive coronary microvascular response in HF. Their pharmacological targeting might improve cardiac function via microvasculature-dependent mechanisms.

Here, we were not able to indicate the specific cardiac cell type of cardiac transcriptome changes due to the use of total cardiac cellular composition for transcriptomic analyses. Nevertheless, we suppose that some transcriptomic changes observed in our study may be related to coronary endothelial cells because they account for approximately 45% of total cardiac cellular composition [110]. Further studies are required to indicate a main cellular source of the observed changes in the cardiac transcriptome within all types of cells forming cardiac tissue.

In sum, in the present work, we have shown that Tgαq*44 mice display accelerated cardiac ageing that is exemplified by robust perivascular fibrosis and extracellular matrix remodelling in coronary microcirculation resulting in CMD. Quite surprisingly, Tgαq*44 mice displaying the systolic-diastolic nature of HF [41] represent an interesting model of altered microvasculature in HF, underscoring the fact that the alterations in coronary microvasculature are not limited to HFpEF [9] or to metabolic derangements [111]. CMD in Tgαq*44 mice is clearly not a primary cause of cardiac pathology in this model but may contribute to the progression of HF that is initially driven by cardiomyocyte-specific overexpression of active Gαq protein [40].

Further studies on interactions between cardiomyocytes and endothelial cells based on angiocrine signals from cardiomyocytes [112, 113], coronary endothelial cell-driven regulation of cardiac contractility [114] or coronary endothelial-dependent regulation of cardiomyocyte metabolism [115, 116] and their contribution to adaptive and maladaptive phases of HF are still required to better understand the molecular mechanisms by which CMD contributes to HF pathophysiology.

Nevertheless, reverting CMD with its accelerated age-related changes in the microvasculature during HF represents a novel target for HF therapy that is not efficiently targeted by current anti-HF therapy, including neurohormonal blockade—the mainstay of HF pharmacotherapy. It is hoped that some of the recently proposed novel therapeutic strategies reversing or inhibiting the fibrotic process in the heart [117] or other microvasculature-targeted approaches [118] could be effective, and Tgαq*44 mice may be a valuable murine model to test their therapeutic efficacy in HF.

## Supplementary Information

Below is the link to the electronic supplementary material.

Supplementary file1 (XLSX 1269 KB)

Supplementary file2 (DOCX 6354 KB)

## Data Availability

The data discussed in this publication have been deposited in NCBI's Gene Expression Omnibus [119] and are accessible through GEO Series accession number GSE207648 (https://www.ncbi.nlm.nih.gov/geo/query/acc.cgi?acc=GSE207648).
