# Supplementary material for: Accelerated ageing and coronary microvascular dysfunction in chronic heart failure in Tgαq*44 mice
Source: GeroScience. 2023 Jan 24;45(3):1619–48. doi: 10.1007/s11357-022-00716-y (PMC10400753; doi:10.1007/s11357-022-00716-y)
Supplement: Supplementary file 2 — Supplementary file2 (DOCX 6354 KB) [file 11357_2022_716_MOESM2_ESM.docx]

Accelerated ageing and coronary microvascular dysfunction in chronic heart failure in Tgαq*44 mice

Journal: GeroScience

Piotr Berkowicz^1^, Justyna Totoń-Żurańska^2^, Grzegorz Kwiatkowski^1^, Agnieszka Jasztal^1^, Tamás Csípő^3,4^, Kamil Kus^1^, Urszula Tyrankiewicz^1^, Anna Orzyłowska^5^, Paweł Wołkow^2^, Attila Tóth^3^, Stefan Chlopicki^1,6^

^1^ Jagiellonian University, Jagiellonian Centre for Experimental Therapeutics (JCET), Krakow, Poland

^2^Centre for Medical Genomics OMICRON, Jagiellonian University Medical College, Krakow, Poland

^3^Division of Clinical Physiology, Department of Cardiology, Faculty of Medicine, University of Debrecen, Debrecen, Hungary

^4^Department of Public Health, Faculty of Medicine, Semmelweis University, Budapest, Hungary

^5^Department of Neurosurgery and Paediatric Neurosurgery, Medical University of Lublin, Lublin, Poland

^6^Jagiellonian University Medical College, Faculty of Medicine, Chair of Pharmacology, Krakow, Poland

**Address for correspondence**:

Prof. Stefan Chlopicki, M.D., Ph.D., Director of JCET

Jagiellonian Centre for Experimental Therapeutics (JCET)

ul. Bobrzyńskiego 14, 30-348 Kraków, Poland

Correspondence e-mail: **stefan.chlopicki@jcet.eu**

**Supplementary materials**

# Supplementary Tables

**Supplementary Table S1 Analytical parameters of the SteroIDQ® kit**

17 analytes and deuterium-labeled internal standards pairs, MRM of analytes, analyte retention times, calibration ranges. Legend: E1 – estrone, E2 – estradiol, DHEA – dehydroepiandrosterone, DHEA-S - dehydroepiandrosterone sulphate, 17OHP - 17-OH-progesterone

| No. | Analyte | Internal standard | Analyte MRM | Retention time [min] | Calibration range [ng/mL] |
| --- | --- | --- | --- | --- | --- |
| 1 | Aldosterone | d7-aldosterone | 361.2/343.2 | 1.77 | 0.05-5 |
| 2 | Androstenedione | d3-androstenedione | 287.2/97.1 | 6.74 | 0.03–8 |
| 3 | Androsterone | d4-androsterone | 273.2/255.1 | 8.05 | 0.06–6 |
| 4 | Corticosterone | d8-corticosterone | 347.2/329.1 | 4.39 | 0.03–30 |
| 5 | Cortisol | d4-cortisol | 363.2/345.1 | 2.34 | 1–1000 |
| 6 | Cortisone | d7-cortisone | 361.2/163.1 | 2.37 | 0.10–100 |
| 7 | 11-Deoxycorticosterone | d8-17OHP | 331.2/109.1 | 6.49 | 0.03–15 |
| 8 | 11-Deoxycortisol | d5-11-deoxycortisol | 347.2/109.1 | 4.75 | 0.01–10 |
| 9 | DHEA | d4-E1 | 271.2/253.2 | 6.75 | 0.12–30 |
| 10 | DHEAS | d5-DHEAS | 271.2/253.2 | 2.78 | 32–8000 |
| 11 | E2 | d3-E2 | 255.2/159.1 | 5.84 | 0.02–20 |
| 12 | E1 | d4-E1 | 271.2/253.2 | 6.62 | 0.03–15 |
| 13 | Etiocholanolone | d4-androsterone | 273.2/255.1 | 7.86 | 0.06–6 |
| 14 | 17OHP | d8-17OHP | 331.2/109.1 | 6.96 | 0.05–50 |
| 15 | Progesterone | d9-progesterone | 315.2/109.1 | 8.46 | 0.06–15 |
| 16 | Testosterone | d5-testosterone | 289.2/97.0 | 6.19 | 0.01–10 |
| 17 | Dihydrotestosterone | d3-dihydrotestosterone | 291.1/255.2 | 7.56 | 0.012–3 |

**Supplementary Table S2. Blood cell count in the course of HF development in Tgαq*44 mice and during ageing process in FVB mice**

**Supplementary Table S3. Differentially expressed genes in FVB *vs.* FVB analysis**

**Supplementary Table S4. Annotated DEGs to the respective over-represented biological processes in FVB *vs.* FVB analysis**

**Supplementary Table S5. Over-represented biological processes from Gene Ontology database in FVB *vs.* FVB analysis**

**Supplementary Table S6. 454 ‘*genes of aged heart’* and 56 ‘*processes of aged heart*’**

**Supplementary Table S7. 481 ‘*genes of ageing heart*’ and 55 ‘*processes of ageing heart*’**

**Supplementary Table S8. Differentially expressed genes in Tgαq*44 *vs.* FVB analysis**

**Supplementary Table S9. Annotated DEGs to the respective over-represented biological processes in Tgαq*44 *vs.* FVB analysis**

**Supplementary Table S10. Over-represented biological processes from Gene Ontology database in Tgαq*44 *vs.* FVB analysis**

**Supplementary Table S11.** **156 genes that were differentially expressed in all age groups in Tgαq*44 *vs.* FVB analysis**

**Supplementary Table S12. ‘*Cardiac ageing genes*’ and ‘*cardiac ageing processes*’ in respective age groups in Tgαq*44 *vs.* FVB analysis**

**Supplementary Table S13. 8 ‘*genes of aged heart*’ activated in cardiac transcriptome of Tgαq*44 mice from early until end-stage HF**

**Supplementary Table S14. 26 ‘*genes of aging heart*’ activated in cardiac transcriptome of Tgαq*44 mice from early until end-stage HF**

# Supplementary Figures

**
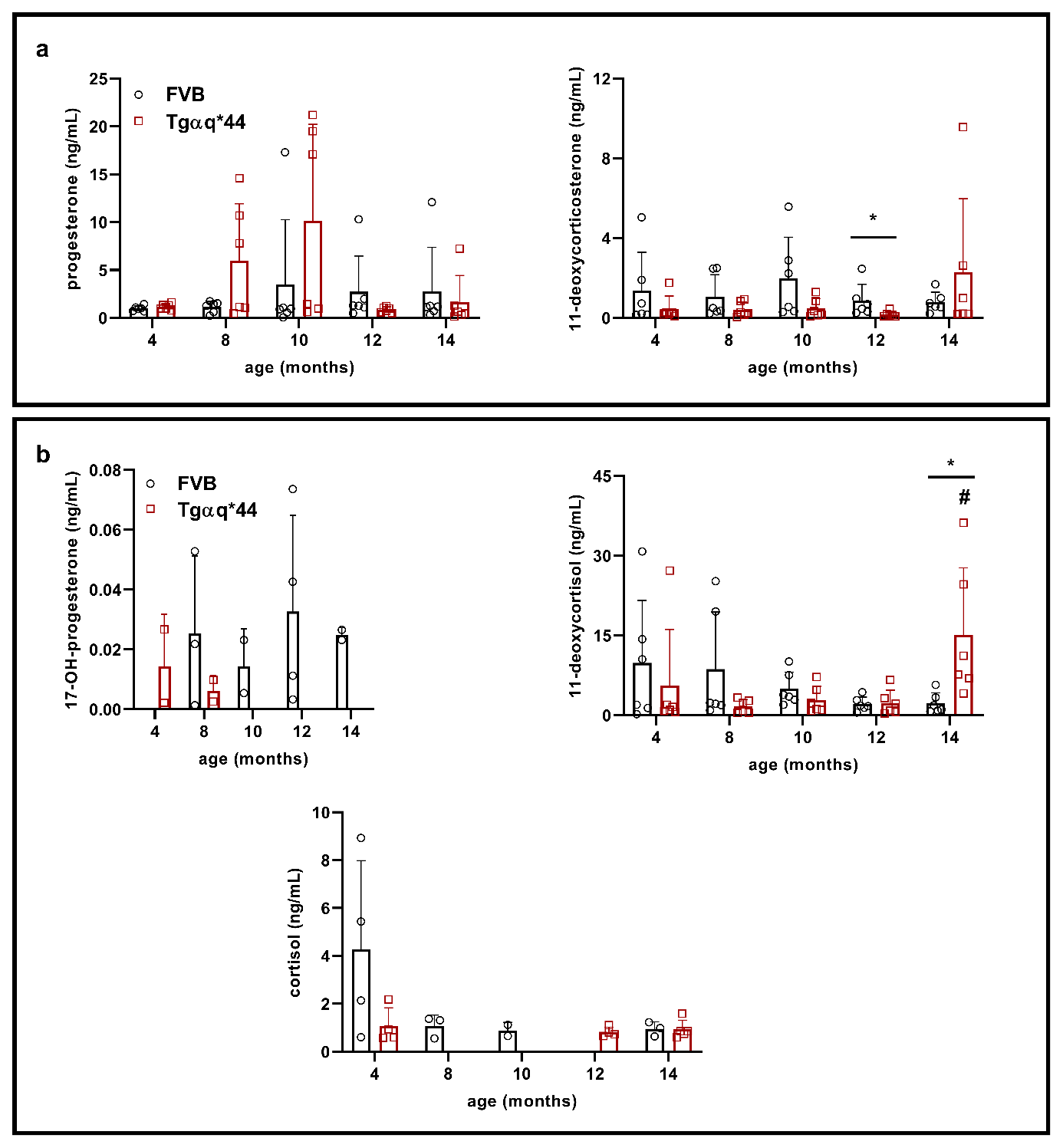
**

**
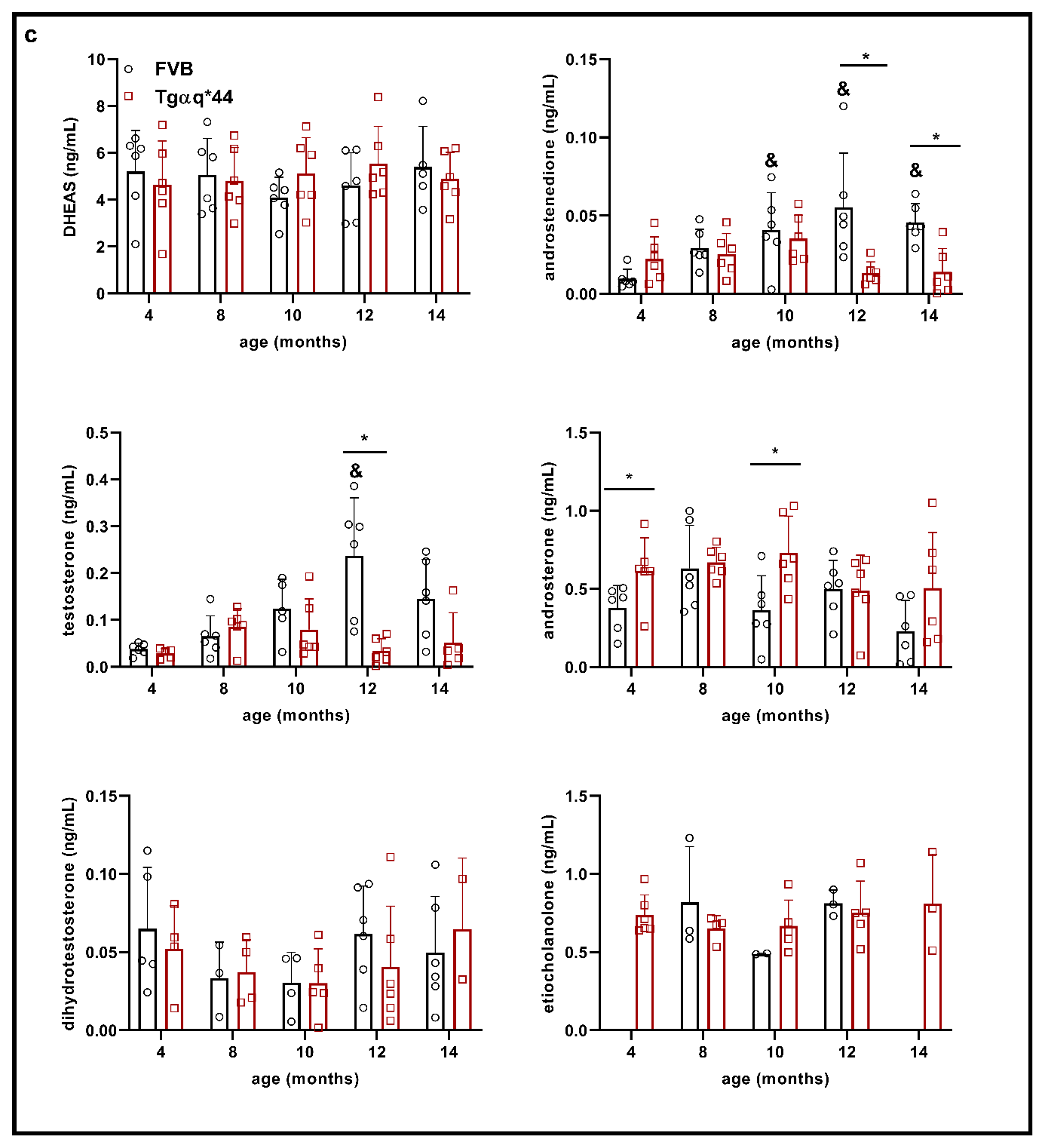
**

**Fig. S1 Profile of steroid hormones in plasma in the course of HF development in Tgαq*44 mice compared with age-related changes in FVB mice.** Concentration of mineralocorticoids **(a)**, glicocortycosteroids **(b)**, androgens **(c)** in plasma of Tgαq*44 and age-matched FVB mice. The data are presented as the mean ± SD; *n* = 2–6, **P* < 0.05 for Tgαq*44 mice vs. age-matched FVB mice (Student’s *t* test or Mann–Whitney); ^#^*P* < 0.05 for older Tgαq*44 mice vs. 4-month-old Tgαq*44 mice; ^&^*P* < 0.05 for older FVB mice vs. 4-month-old FVB mice (one-way ANOVA with post hoc Tukey’s test or Kruskal–Wallis test with post hoc Dunn’s test). Legend: DHEA-S - dehydroepiandrosterone sulphate

**
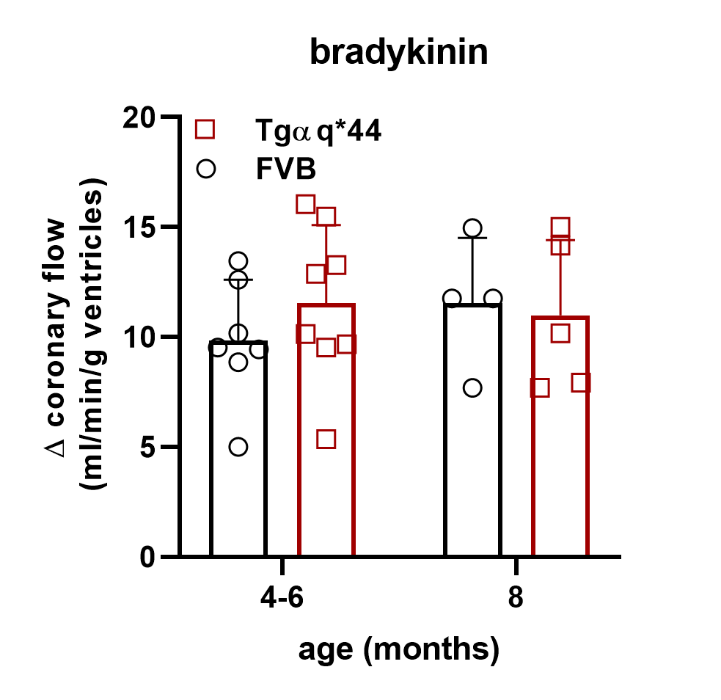
**

**Fig. S2 Coronary vasodilation in response to bradykinin in 4**– to **6- and 8-month-old Tgαq*44 mice compared to age-matched FVB mice.** Results for increase in coronary flow in response to bradykinin (bolus 10 nmol) in isolated perfused murine hearts according to Langendorff method. The data are presented as the mean ± SD; *n* = 4–8, Tgαq*44 mice vs*.* age-matched FVB mice (Student’s *t* test)

**
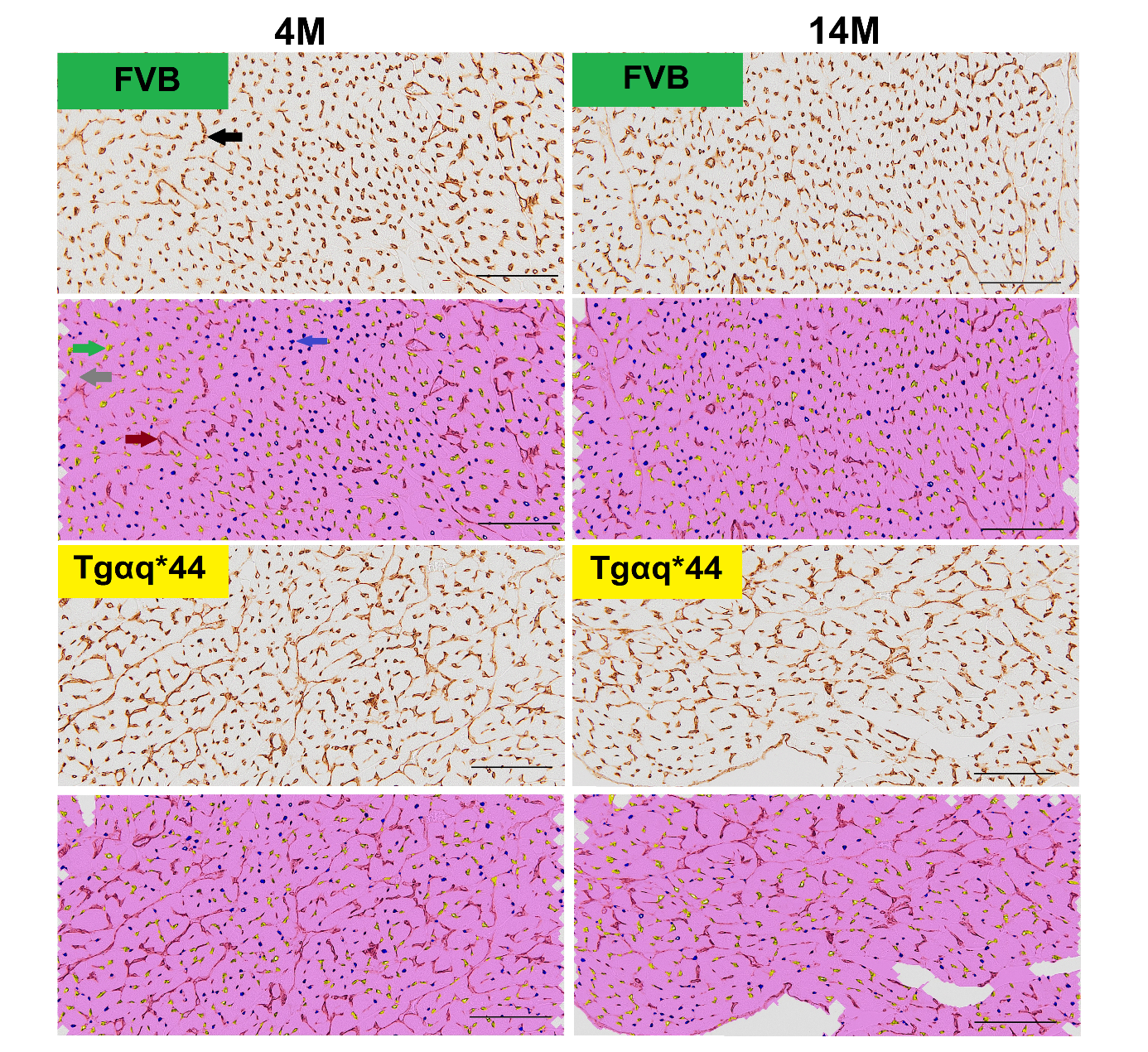
Fig. S3 Representative images of cardiac tissue of 4- and 14-month-old Tgαq*44 mice and age-matched FVB mice illustrating cardiac capillary arrangement.** Cardiac tissue was stained with lectin and DAB. Images of cardiac tissue stainings without (upper panel) and with (lower panel) applying an algorithm (scale bars indicate 100 µm – × 200 magnification); upper panel: capillaries (black arrow); lower panel: myocardium (grey arrow), capillaries; area of longitudinal microvessels (brown arrow), area of oblique microvessels (green arrow), area of transverse microvessels (navy blue arrow). Legend: 4M–4 months of age, 14M–14 months of age


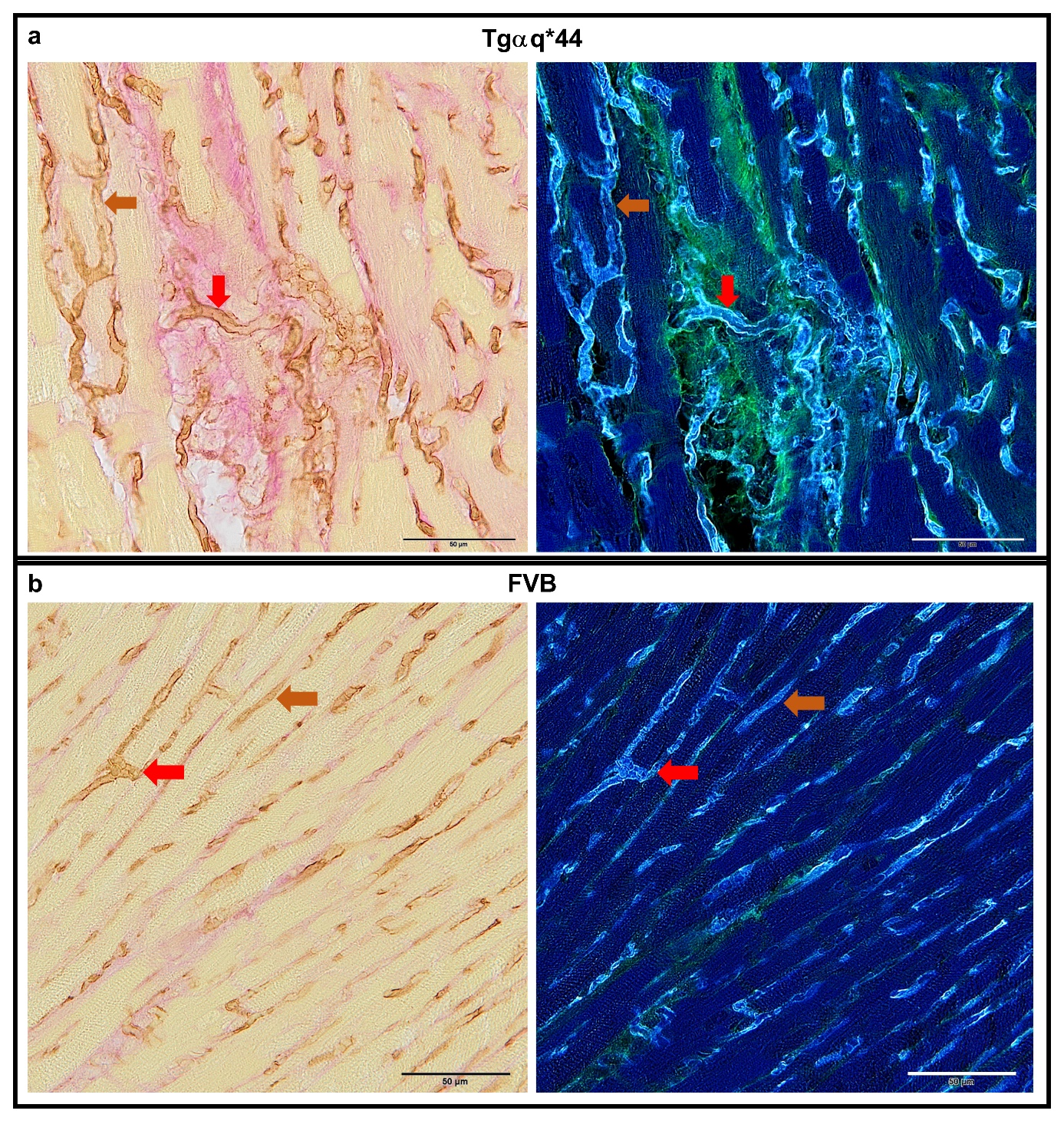


**Fig. S4 Cardiac capillary arrangement in 14-month-old Tgαq*44 and age-matched FVB mice.** Representative images of cardiac tissue illustrating cardiac capillary arrangement in longitudinal-sections of the free wall of the left ventricle in Tgαq*44 (**a**) and FVB mice (**b**). Cardiac tissue was stained with: lectin and DAB (left panel); lectin, PAS and PSR (right panel). Scale bars indicate 50 µm – × 400 magnification. Capillaries that go along (brown arrows) and across (red arrows) cardiomyocytes in cardiac muscle fibre are depicted as oblique and transverse microvessels and longitudinal microvessels in Fig. 3, S3, respectively.


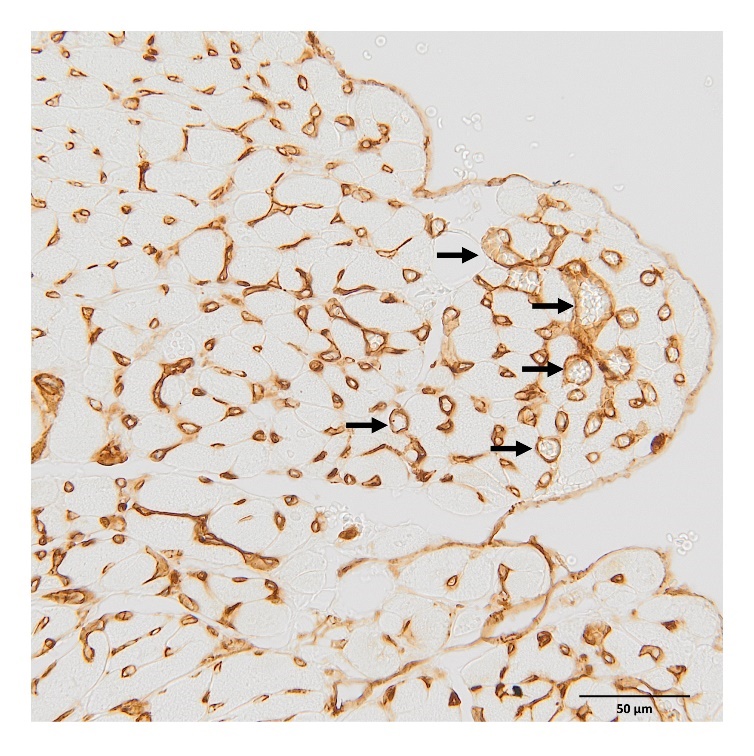


**Fig. S5 Representative image of cardiac tissue of 14-month-old Tgαq*44 mice illustrating capillaries’ lumen enlargement in cross-section of the papillary muscle of the left ventricle.** Cardiac tissue was stained with lectin. Scale bars indicate 50 µm – × 400 magnification. Capillaries’ lumen enlargement is indicated by black arrows.

| **a**  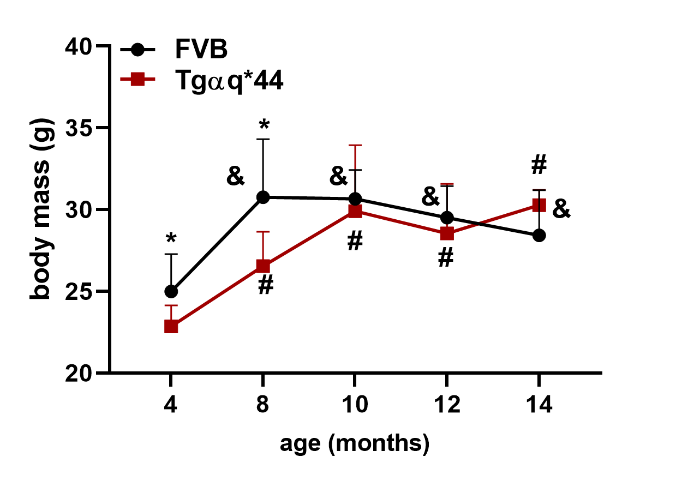 | **b**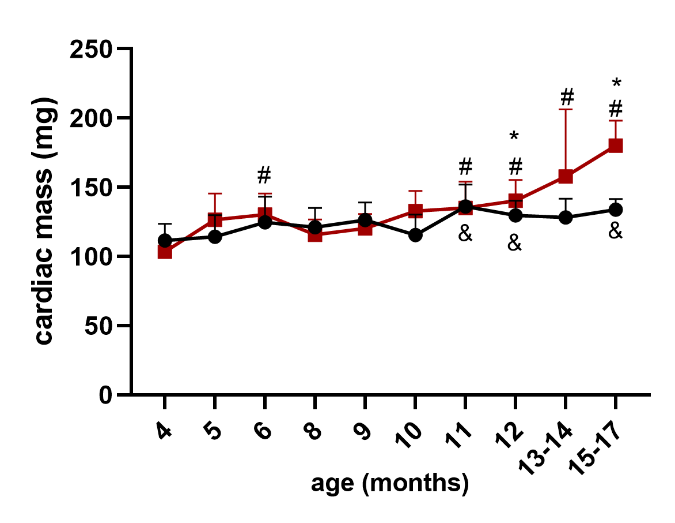 |
| --- | --- |
| **c**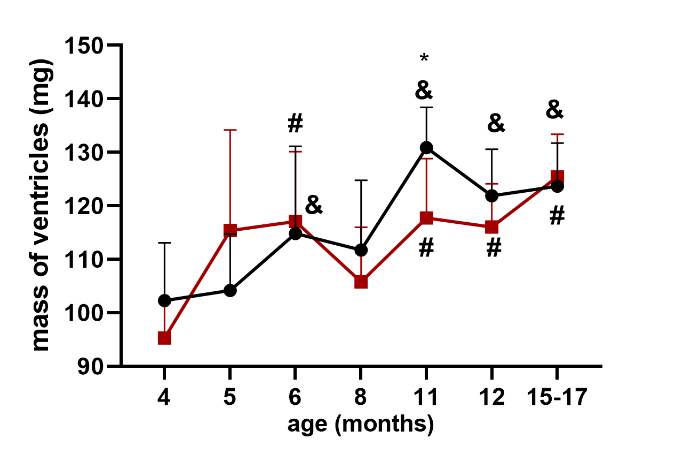 | **d**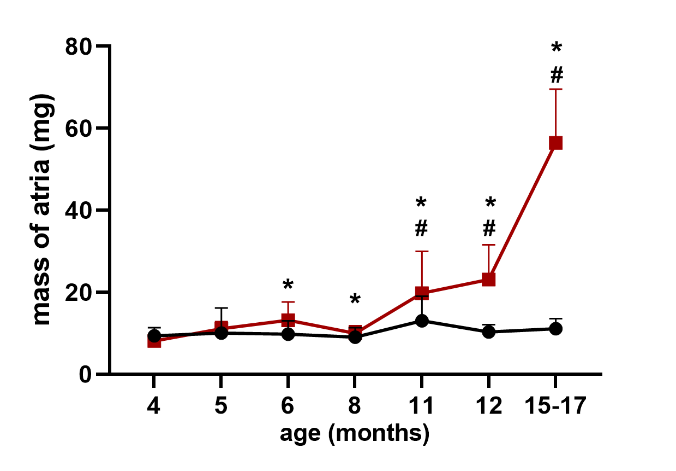 |

**Fig. S6 Changes in body (a), heart (b), ventricles (c), atria (d) mass in the course of HF development in Tgαq*44 mice compared to age-matched FVB mice.** The data are presented as the mean ± SD; *n* = 3–37, **P* < 0.05 for Tgαq*44 mice vs. age-matched FVB mice (Student’s *t* test or Mann–Whitney); ^#^*P* < 0.05 for older Tgαq*44 mice vs. 4-month-old Tgαq*44 mice; ^&^*P* < 0.05 for older FVB mice vs. 4-month-old FVB mice (one-way ANOVA with post hoc Tukey’s test or Kruskal-Wallis test with post hoc Dunn’s test)

**
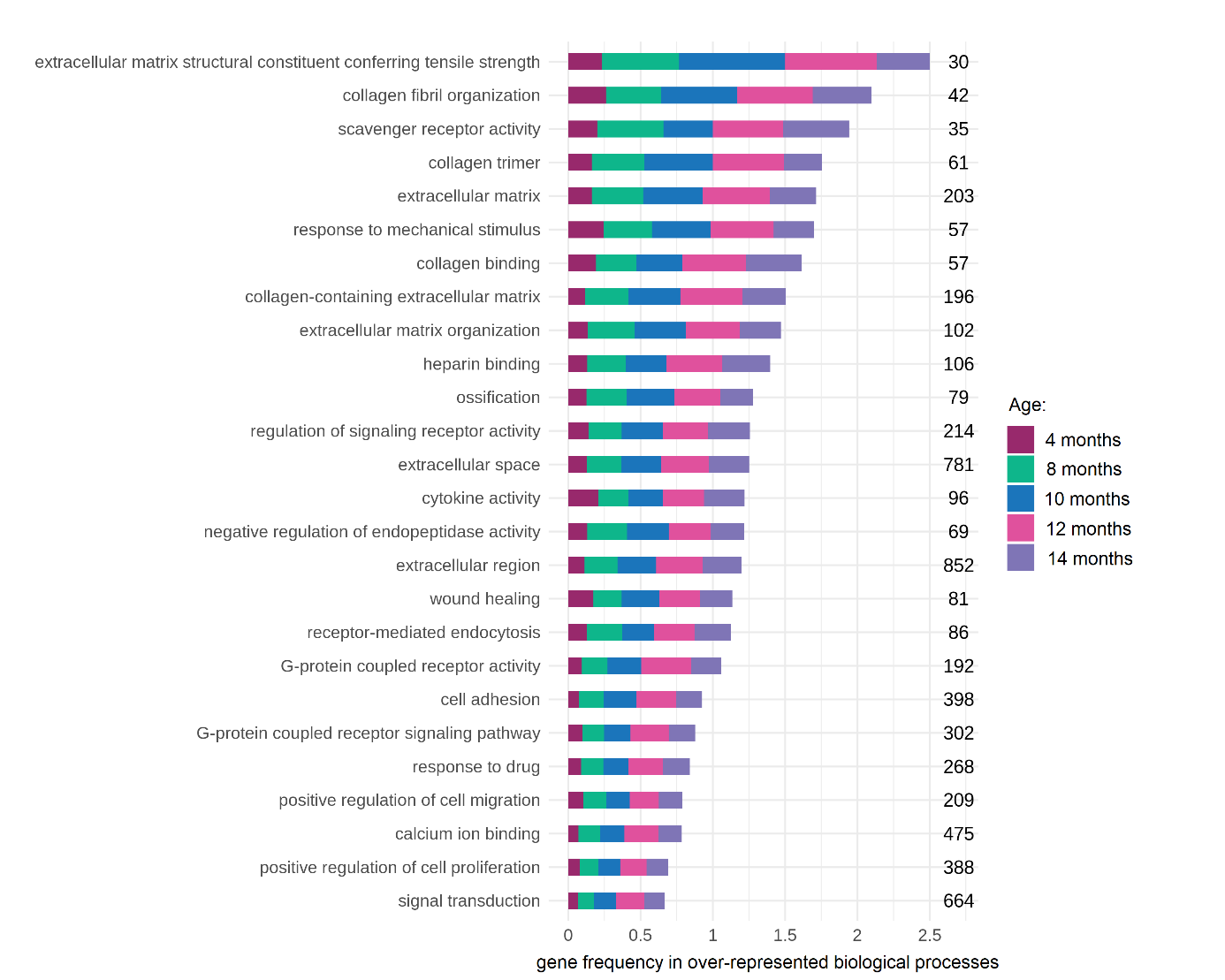
**

**Fig. S7 26 biological processes that were over-represented in all age groups in Tgαq*44 vs. FVB analysis.** Diagram presents gene frequency of DEGs in 26 over-represented biological processes in all age groups in Tgαq*44 *vs.* FVB analysis. 8 out of 26 over-represented biological processes in all age groups in Tgαq*44 vs*.* FVB analysis represent „*processes of ageing heart*” and are presented in Fig. 9
